# Structure optimization of new tumor-selective Passerini α-acyloxy carboxamides as Caspase-3/7 activators

**DOI:** 10.1038/s41598-022-26469-4

**Published:** 2022-12-27

**Authors:** Mohammed Salah Ayoup, Yasmin Wahby, Hamida Abdel-Hamid, Marwa M. Abu-Serie, Mohamed Teleb

**Affiliations:** 1 Chemistry Department, Faculty of Science, Alexandria University, P.O. Box 426, Alexandria, 21321 Egypt; 2 Medical Biotechnology Department, Genetic Engineering and Biotechnology Research Institute, City of Scientific Research and Technological Applications (SRTA-City), Borg El Arab, Egypt; 3 Department of Pharmaceutical Chemistry, Faculty of Pharmacy, Alexandria University, Alexandria, 21521 Egypt

**Keywords:** Medicinal chemistry, Organic chemistry

## Abstract

Selective elimination of tumors has always been the mainstay of oncology research. The on-going research underlying the cellular apoptotic mechanisms reveal caspases activation, especially the key effector caspase-3, as a personalized tumor-selective therapeutic strategy. Our continued research protocol has exploited new optimized Passerini α-acyloxy carboxamides as efficient apoptotic inducers via caspase-3/7 dependent mechanism with highly selective anticancer profiles. The adopted design rationale relied on excluding structural alerts of previous leads, while merging various pharmacophoric motifs of natural and synthetic caspase activators via optimized one-pot Passerini reaction conditions. The prepared compounds resulting from Passerini reaction were screened for their cytotoxic activities against colorectal Caco-2 and liver HepG-2 cancer cells compared to normal fibroblasts utilizing MTT assay. Notably, all compounds exhibited promising low-range submicromolar IC_50_ against the studied cancer cell lines, with outstanding tumor selectivity (SI values up to 266). Hence, they were superior to 5-fluorouracil. Notably, **7a, 7g**, and **7j** conferred the highest potencies against Caco-2 and HepG-2 cells and were selected for further mechanistic studies**.** Caspas-3/7 activation assay of the hit compounds and flow cytometric analysis of the treated apoptotic cancer cells demonstrated their significant caspase activation potential (up to 4.2 folds) and apoptotic induction capacities (up to 58.7%). Further assessment of Bcl2 expression was performed being a physiological caspase-3 substrate. Herein, the three studied Passerini adducts were able to downregulate Bcl2 in the treated Caco-2 cells. Importantly, the mechanistic studies results of the three hits echoed their preliminary MTT antiproliferative potencies data highlighting their caspase-3 dependent apoptotic induction. Finally, the in silico predicted physicochemical and pharmacokinetic profiles, as well as ligand efficiency metrics were drug-like.

## Introduction

**Figure 1:**
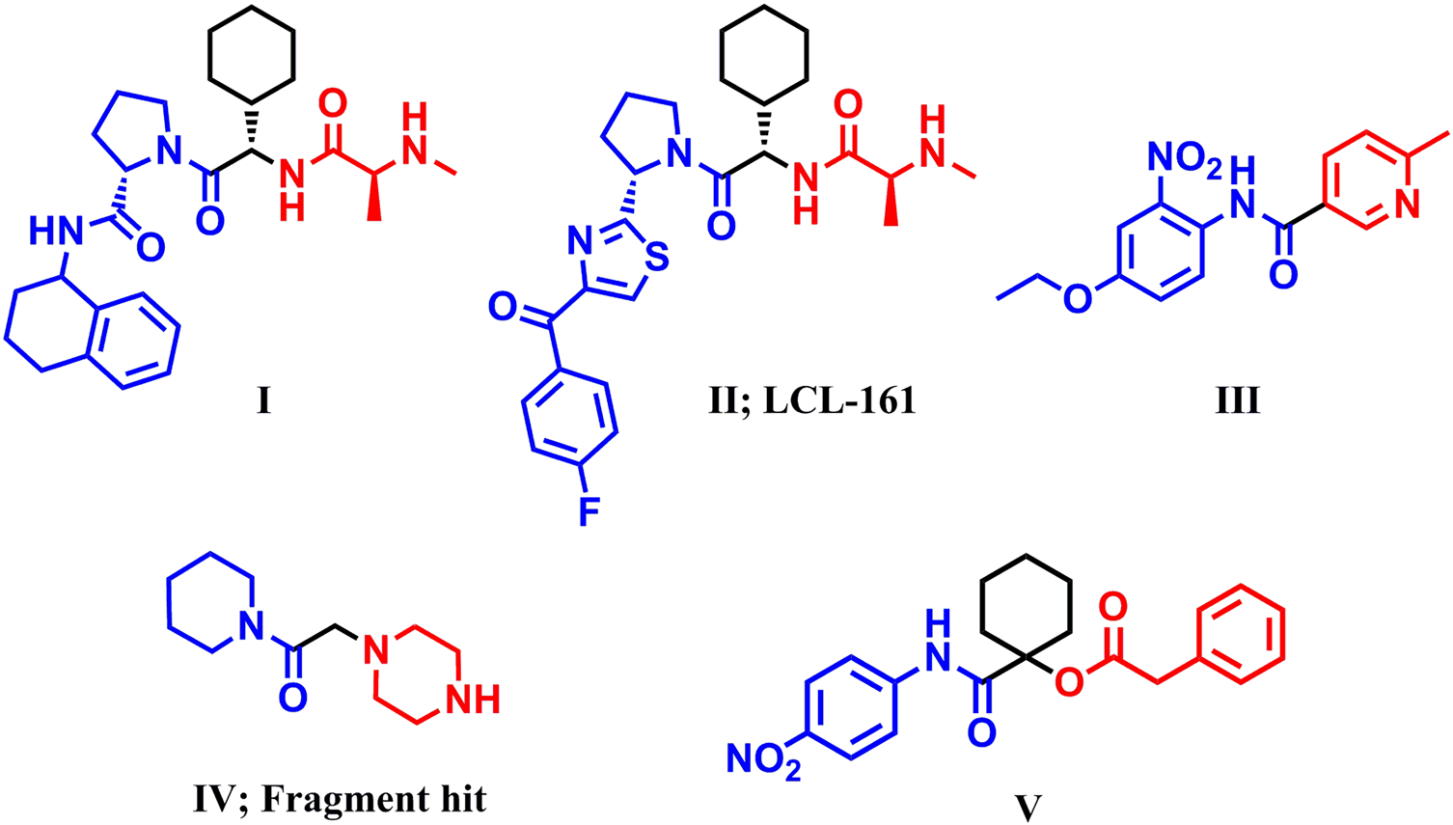
Lead Small-molecules caspase activators.

Evading apoptosis, the normal programmed cell death process that maintains tissue homeostasis^1^, is a cancer hallmark^2,3^. Apoptosis is mediated through a cascade of intrinsic and extrinsic signaling pathways that mainly converge on caspase-dependent proteolysis of numerous vital proteins, DNA cleavage, and membrane blebbing^4^. Caspases comprise a characteristic family of cysteinyl-aspartate-specific proteases that are capable of cleaving an aspartate amino acid residue from their specific substrates^5,6^ inducing irreversible cell death. Caspases are classified into initiator (caspase-10, -9, -8 and -2) and effector (caspase-7, -6 and -3) groups^7^. Caspase-3 and -7 are considered the key effector caspases executing apoptosis^8–10^. These findings thus directed cancer research programs to set the caspases family, especially the effector members, as attractive anticancer targets for inducing apoptosis induction in cancer cells^11^. Moreover, the cellular levels of procaspases, the inactive precursors of effector caspases, are usually elevated in cancer relative to normal tissue, introducing an ideal opportunity for tumor-targeting to selectively generate cytotoxic caspases in cancer through weaponizing procaspases overexpression. Accordingly, caspase-mediated apoptotic induction is considered a highly tumor-selective anticancer strategy^12^. Since mature caspases are inhibited using the cellular inhibitor of apoptosis proteins (IAPs) that are essentially inactivated by the secondary mitochondria-derived activator of caspases (SMAC/Diablo)^7^, early drug discovery trials have focused on SMAC that triggers caspase-dependent apoptosis^13,14^. However, its direct clinical application was hampered by the drawbacks of its peptide nature. A tetrapeptide of SMAC was then envisioned as the principal motif for designing peptidomimetics that can function as the endogenous protein. Pioneer studies introduced series of efficient apoptotic inducers designed as capped tripeptides to reduce their peptide character (**Fig. 1**; **I** and **II**)^16^. This active research was mirrored by several trials to optimize novel small-molecule caspase-dependent apoptotic inducers. Early studies employing high throughput screening caspase-based assays introduced a new series of potent caspase activators derived from nicotinamides **III** (Fig. 1)^16^. Further fruitful work led to various preclinical-stage caspase activators have been introduced^17,18^. Optimized fragment-based design studies identified nonpeptidic clinical-stage apoptotic inducers that are based on an essential amide core (**Fig. 1**, **IV**) that is crucial for activity and should not be changed or removed^19^. Along this line, our research group have utilized the multicomponent Passerini-3CR as a facial synthetic strategy to synthesize libraries of amide-based caspase activators inducers **V** (Fig. 1)^20^ incorporating the thematic structural features of lead compounds (Fig. 1). Among the evaluated series, the hit derivatives exerted profound anticancer potency against various human cancers^20^.

## Rationale design

**Figure 2:**
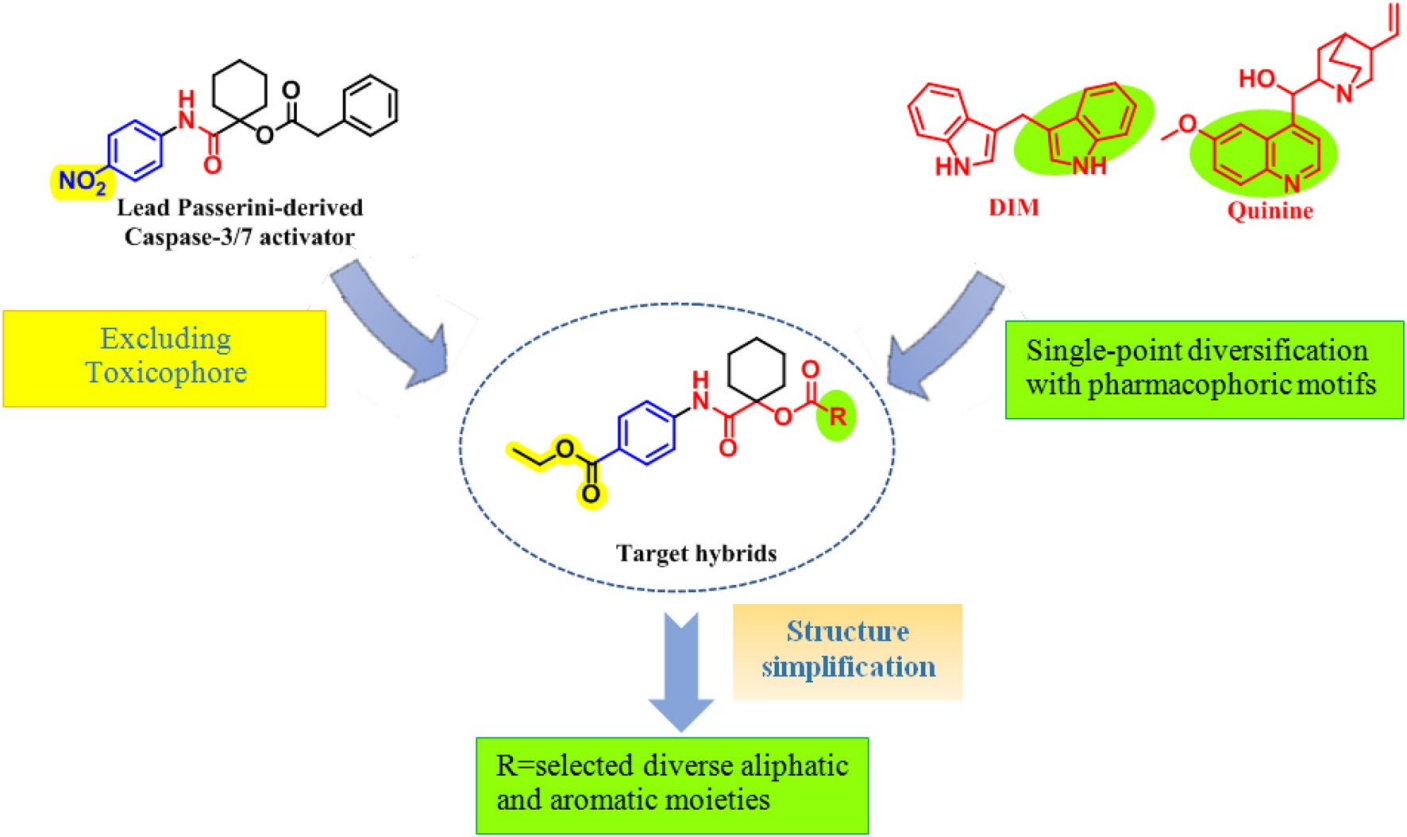
Design rationale of the target Passerini adducts.

Herein, we continued our optimization protocol utilizing the lead Passerini scaffold (Fig. 2). Firstly, the newly synthesized α-acyloxy carboxamides were designed to incorporate an ester to the amide counterpart as a substitute to the nitro group being a structural alert and a possible toxicophore^21,22^ aiming to enhancing the molecule’s safety profile and anticancer selectivity. Secondly, the acyloxy counterpart was rationally selected inspired by pharmacophoric motifs of natural caspase activators, where indole and quinoline rings were installed to the skeleton being the main cores of marine bisindole alkaloids^23,24^ from the 3,3′-diindolylmethane (DIM) family^25^ and quinoline alkaloids^23^. Finally, to possibly limit “the molecular obesity” for better pharmacokinetics as well as to enrich the deduced SAR in this study, structure simplification strategy was adopted, where the heterocyclic acyloxy counterparts were simplified to diverse aromatic (benzoate, phenylacetate, and cinnamate) and aliphatic (thioglycolate) moieties. As part of the followed mimicry design approach, various substituents that conferred caspase-dependent antitumor activities to reported lead molecules were selected including halogens^20^, and thiol^26^ group.

All the target Passerini adducts were initially screened via the MTT assay^27^ against human fibroblasts Wi-38 for their cytotoxic potential against human fibroblasts (Wi-38) and two cancer cell lines namely HepG-2 (liver) and Caco-2 (colon) being among the most common cancers worldwide according to the updated WHO list^28^. The selectivity against the cancer versus normal cells were evaluated. Activation assay of caspase-3/7 and flow cytometric analysis of apoptosis were performed to the most promising compounds. Further apoptosis studies were performed to quantify the expression of Bcl2 that is a physiological caspase-3 substrate^29^. Thereafter, the hit compounds were subjected to computational studies to predict their physicochemical properties, ADME parameters and efficiency metrics.

## Results and discussion

### Chemistry

Multicomponent reactions (MCRs) have become excellent processes in organic studies due to their bond formation efficiency since three or more components react together in one-pot synthesis to achieve higher yields of pure products, enhanced reaction rate, better selectivity, improved ease of manipulation, rapid optimization of reaction, ability to effect, chemo-, regio-, and stereoselective transformations, economical and eco-friendly processes. Isocyanide based multicomponent reactions (IMCRs) are one of the most important fields in the last decade. IMCRs represent as interesting close of multicomponent reactions due to their versatile biological properties. Moreover, they were useful for the formation of novel, complex and biological active compounds^30,31^.

Mario Passerini performed the first IMCR by combining three components (an isocyanide, carboxylic acid, and oxo-compound) to form α-acyloxy carboxamides. Passerini products are scaffolds that have been used for synthesizing large libraries of bioactive drugs^32,33^.

Novel synthesized α-acyloxy carboxamides have shown cytotoxic activities with different human cancer cell lines. Three-component Passerini reaction was reported by using *p*-nitrophenyl isocyanide previously, in which the nitro group as an electron withdrawing group with cyclohexanone and different carboxylic acids. Also unexpected products obtained by using trifluoro- and trichloro-acetic acids forming hydroxy and spiro compounds, respectively. In this respect, we report herein the Passerini reaction between ethyl-4-isocyanobenzoate **4** with carboxylic acid derivatives **5a–k** and cyclohexanone **6** forming biologically active products^20^.

**Figure 3:**
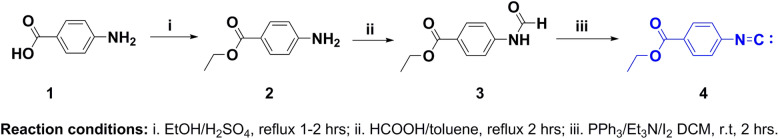
Synthesis of ethyl-4-isocyanobenzoate **4**.

Herein, our strategy to carry out the Passerini reaction utilizing the isocyanide **4**, which was synthesized by performing of esterification reaction of 4-amino benzoic acid (**1**) by reflux in absolute ethanol to afford the corresponding ester 4-amino ethyl benzoate (**2**) which known as benzocaine drug that used as pain reliever^34^. Consequently, formylation of the latter utilizing formic acid and toluene to give 4-formamido ethyl benzoate (**3**)^35^ followed by dehydration using PPh_3_/Et_3_N/I_2_ protocol where the reaction was proceeded for 1 h to afford the corresponding isonitrile ethyl-4-isocyanobenzoate (**4**) in good yield^36^ (Fig. 3). The formed isocyanide is unstable, and it is preferable to store it at a low temperature (5–10 °C). The structure of **4** was confirmed by IR that showed strong band at 2124 and 1717 cm^−1^ that characterizes the isocyano and carbonyl of the ester groups respectively.

**Figure 4:**
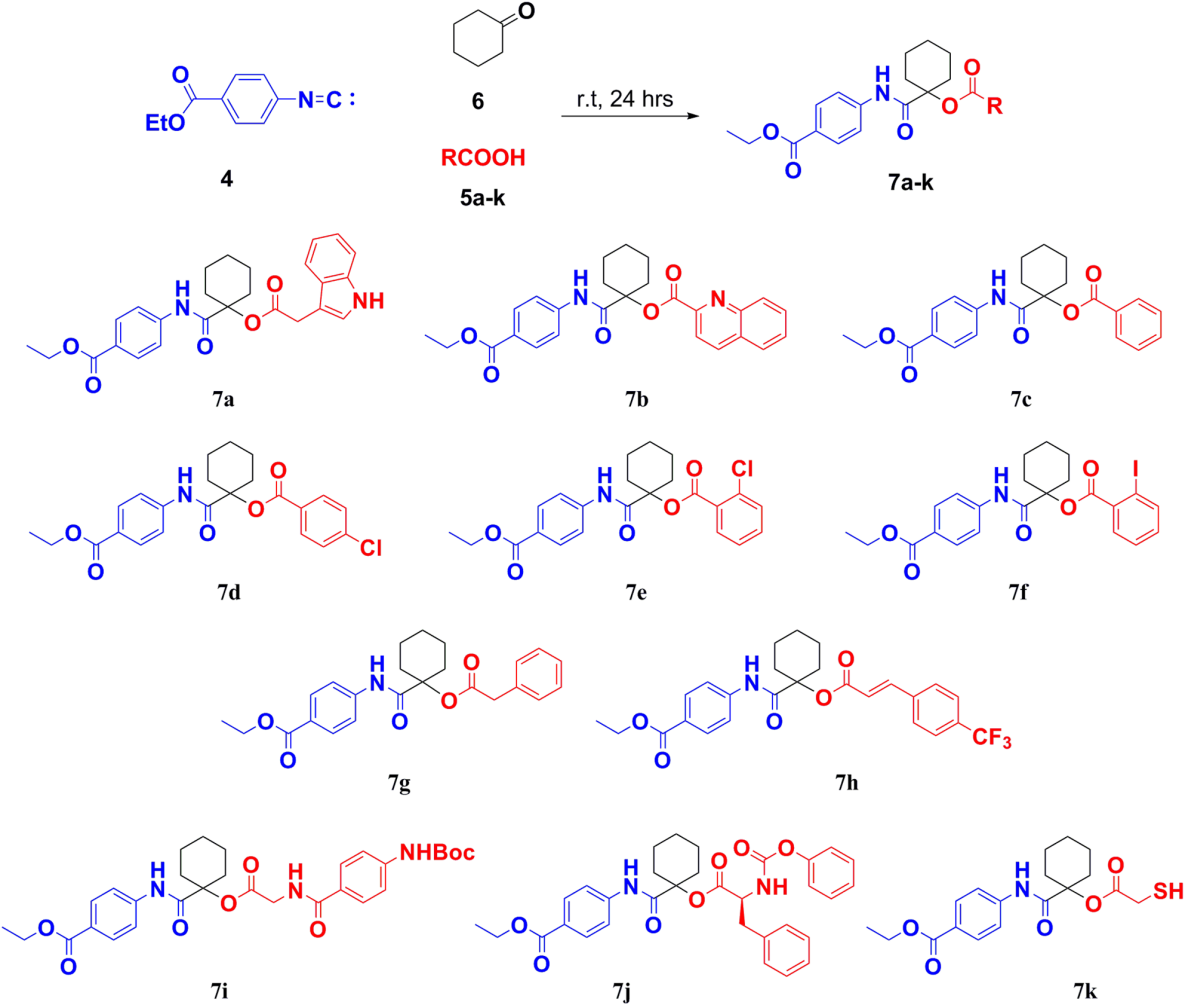
Synthesis of α-acyloxy carboxamide derivatives **7a–k** via Passerini reaction.

**Figure 5:**
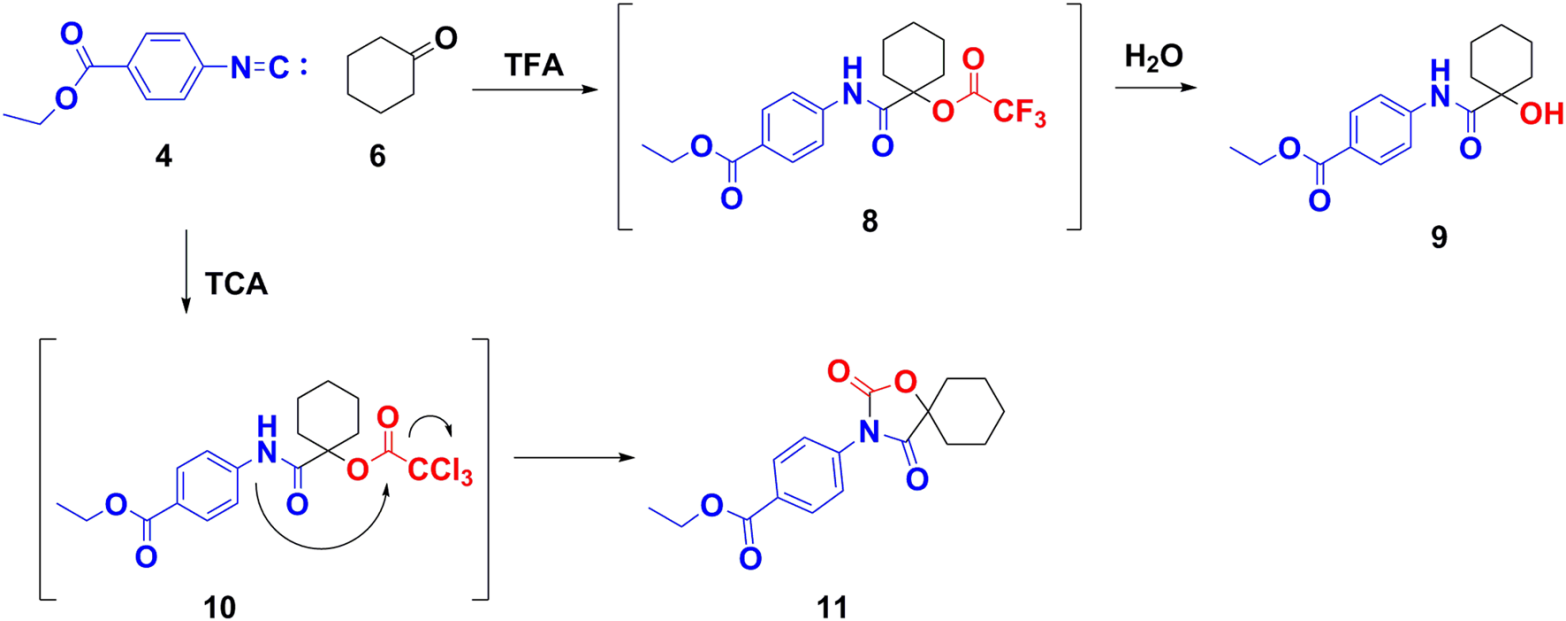
Synthesis of **9** and **11** via Passerini reaction using trihaloacetic acids.

A novel series of the α-acyloxy carboxamide derivatives **7a–k** were prepared via Passerini reaction by using **4** in excess cyclohexanone as a ketone and solvent with miscellaneous carboxylic acids namely indole-3-acetic acid, quinaldic acid, benzoic acid, *p*-chlorobenzoic acid, *o*-chlorobenzoic acid, *o*-iodobenzoic acid, phenylacetic acid, (*E*)-3-(4-(trifluoromethyl)cinnamic acid, *N*-Boc-4-aminohippuric acid, (phenoxycarbonyl)-l-phenylalanine, and 2-mercaptoacetic acid **5a–k**, respectively**;** at room temperature (Fig. 4). The obtained Passerini derivatives were analyzed in detail by FT-IR and NMR spectroscopy. ^1^H-NMR spectrum of **7a–k** showed signals for –NH in amido groups resonating from δ_H_ 10.9–8.9 ppm. The ten Protons of *c*-Hexylidene group also appeared at range from *δ*_*H*_ 3.1–1.1 ppm. The proton of –NH indole group **7a** showed singlet signal at δ_H_ 10.94 ppm. Also, two protons of –CH_2_ group (CO-C**H**_**2**_-Ar) for compound **7g** appeared as singlet signal at δ_H_ 3.78 ppm. Proton spectrum for compound **7h** recorded two signals corresponding to olefinic hydrogens at δ_H_ 7.71 and 6.86 ppm. Three amide protons for product **7i** showed three signals at δ_H_ 9.74, 9.62 and 8.90 ppm. For product **7j**, three protons of l-phenylalanine moiety recorded signals at δ_H_ 4.95 ppm for –CH_2_ and from δ_H_ 4.44–4.39 ppm for –CH. Proton of Mercapto group –SH in **7k** showed signals resonating from δ_H_ 0.85–0.80 ppm. ^13^C-NMR spectrum recorded signals resonated from δ_c_ 171.8–161.2 ppm corresponding to amido and ester CO groups, respectively. Carbons of *c*-Hexylidene (O–**C**–CO) resonating at range from δ_c_ 84.0–81.1 ppm. Also, Cyclohexyl carbons recorded signals resonating from δ_c_ 36.0–20.6 ppm. For products **7f** and **7k**, carbons of **C**-I and **C**H_2_-SH showed signals recorded at δ_c_ 94.7 and 40.7 ppm, respectively.

(P-3CR) utilizing trihaloacetic acids via Passerini reaction showed unexpected derivatives. Where, trifluoroacetic acid (TFA) afforded Ethyl 4-(1-hydroxycyclohexanecarboxamido)benzoate **9** in a good yield after hydrolyzing the formed Passerini product ethyl 4-(1-(2,2,2-trifluoroacetoxy)cyclohexanecarboxamido)benzoate **8** (Fig. 5). ^1^H-NMR spectrum confirmed the structure of **9**, where two protons correspond to amide –NH and hydroxyl group –OH appeared clearly as two singlet signals at δ_H_ 9.91 and 5.49 ppm, respectively. Also, ^13^C-NMR spectrum showed signals for carbonyl of the amide group and carbons of tertiary alcohol at δ_c_ 177.1 and 74.5 ppm, respectively.

Alternatively, P-3CR using trichloroacetic acid (TCA) afforded the unexpected spiro compound; Ethyl 4-(2,4-dioxo-1-oxa-3-azaspiro[4.5]decan-3-yl)benzoate (**11)**, where Passerini product; ethyl 4-(1-(2,2,2-trichloroacetoxy)cyclohexanecarboxamido)benzoate **10** was formed first, then releasing of –CCl_3_ group through intramolecular nucleophilic nitrogen attack on trichloro acetyl group, followed by cyclization to form **11** (Fig. 5). ^1^H-NMR approved the structure of **11**, where protons of aromatic moiety recorded two obvious doublets signals at δ_H_ 8.06 and 7.62 ppm. Moreover, *c*-Hexylidene protons signals appeared at δ_H_ 2.04 and 1.30 ppm. ^13^C-NMR confirmed the spiro carbon signal at δ_c_ 85.0 ppm, Also, CO groups of oxazolinedione ring recorded two signals at *δ*_*c*_ 174.0 and 152.8 ppm.

**Figure 6:**
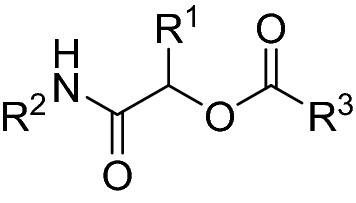
The general structure of Passerini α-acyloxy carboxamides.

Regarding the stability of the currently studied Passerini adducts, literature review revealed a recent valuable study demonstrating that the stability of the ester in the Passerini skeleton could be influenced by other substructures present based on experiments carried on a library of more than Passerini adducts^37^. Being concerned with the medicinal chemistry applications of Passerini reaction, the study focused on investigating the metabolic stability of the scaffold towards esterases. Accordingly, when the group directly attached to the ester moiety (R^3^; Fig. 6) is an ortho-substituted or ortho, ortho′-disubstituted aromatic ring, the Passerini α-acyloxy carboxamides are metabolically stable to esterases^37^.

This is in accordance with previous studies that generally showed that steric hindrance can reduce or suppress the hydrolytic activity of esterases, while the presence of electron-withdrawing groups on the acyl side can facilitate the enzymatic cleavage^38^. The skeleton ester is also stable toward hydrolysis when the R^1^ group is a bulky substituent regardless of the nature of the R^3^ substituent. The study was also pointed to the stability of the terminal aromatic ester as exemplified by experimental observation of the different hydrolytic stabilities of methyl benzoate and methyl 2,6-dimethylbenzoate toward esterases, where the former is readily hydrolyzed, while the latter is completely resistant to esterases^37^.

### Biology

#### Cytotoxicity screening

The newly synthesized Passerini adducts **7a–k** were preliminarily screened utilizing the microculture MTT assay^39^ to evaluate their potential antiproliferative activities against normal human fibroblasts (Wi-38), colorectal (Caco-2) and hepatocellular (HepG-2) cancer cells compared to the standard chemotherapy 5-fluorouracil that promote caspase-dependent apoptosis in various cancers^40,41^ (Table 1). Obviously, all derivatives were superior to 5-fluorouracil against the screened cell lines concerning potency and selectivity. **7g** was ranked as the most potent (IC_50_ = 0.06 μM) and selective (SI = 262–266) Passerini adduct against the screened cell lines, followed by **7j** and **7a** (IC_50_ = 0.07–0.12 μM, SI = 97–243)**. 7e**, **7f**, **7i** and **9** exhibited slightly lower potency and recorded comparable low range submicromolar IC_50_ values. These derivatives were notably tumor-selective with SI ranging from 69 to 180. The remainder derivatives were relatively less active, but still promising regarding potency and selectivity.

**Table 1:** In vitro cytotoxicity profiles of the Passerini adducts against Wi-38, Caco-2 and HepG-2 cells in terms of IC_50_ as detected by MTT assay and their selectivity index (SI) values.

Compound No.	Wi-38		Caco-2		HepG-2	
	EC_100_ (µM)	IC_50_ (µM)	IC_50_ (µM)	SI	IC_50_ (µM)	SI
**7a**	**2.981 ± 0.640**	**11.195 ± 3.753**	**0.088 ± 0.010**	**127.215**	**0.115 ± 0.016**	**97.347**
**7b**	1.791 ± 0.111	18.180 ± 2.218	0.172 ± 0.022	105.697	0.228 ± 0.027	79.736
**7c**	2.099 ± 0.092	7.703 ± 0.321	0.276 ± 0.034	27.909	0.184 ± 0.009	41.864
**7d**	1.695 ± 0.165	24.758 ± 10.213	0.201 ± 0.019	123.174	0.407 ± 0.016	60.830
**7e**	1.779 ± 0.169	19.752 ± 1.021	0.117 ± 0.010	168.820	0.130 ± 0.009	151.938
**7f**	0.967 ± 0.026	17.055 ± 1.694	0.129 ± 0.028	132.209	0.147 ± 0.046	116.020
**7g**	**1.299 ± 0.105**	**17.077 ± 2.149**	**0.064 ± 0.006**	**266.828**	**0.065 ± 0.004**	**262.723**
**7h**	1.654 ± 0.039	18.677 ± 2.040	0.138 ± 0.014	135.340	0.259 ± 0.073	72.111
**7i**	1.627 ± 0.259	22.805 ± 3.101	0.132 ± 0.050	172.765	0.127 ± 0.044	179.566
**7j**	**1.768 ± 0.694**	**16.764 ± 0.637**	**0.069 ± 0.006**	**242.956**	**0.087 ± 0.007**	**192.689**
**7k**	1.465 ± 0.010	9.668 ± 0.102	0.140 ± 0.003	69.057	0.141 ± 0.000	68.567
**9**	3.223 ± 0.217	16.828 ± 3.014	0.154 ± 0.007	109.272	0.150 ± 0.004	112.186
**11**	4.943 ± 1.542	9.921 ± 1.608	0.239 ± 0.071	41.510	0.209 ± 0.017	47.468
**Fu**	0.840 ± 0.055	1.990 ± 0.1353	0.865 ± 0.039	2.301	1.613 ± 0.028	1.233

Significant values are in bold.

*Values are expressed as mean ± SEM. SI is the ratio between the compound’s IC_50_ against normal cells and its IC_50_ against cancer cells. SI ≥ 3 refers to selective compound [30]. 5-FU stands for 5-fluorouracil.

**Figure 7:**
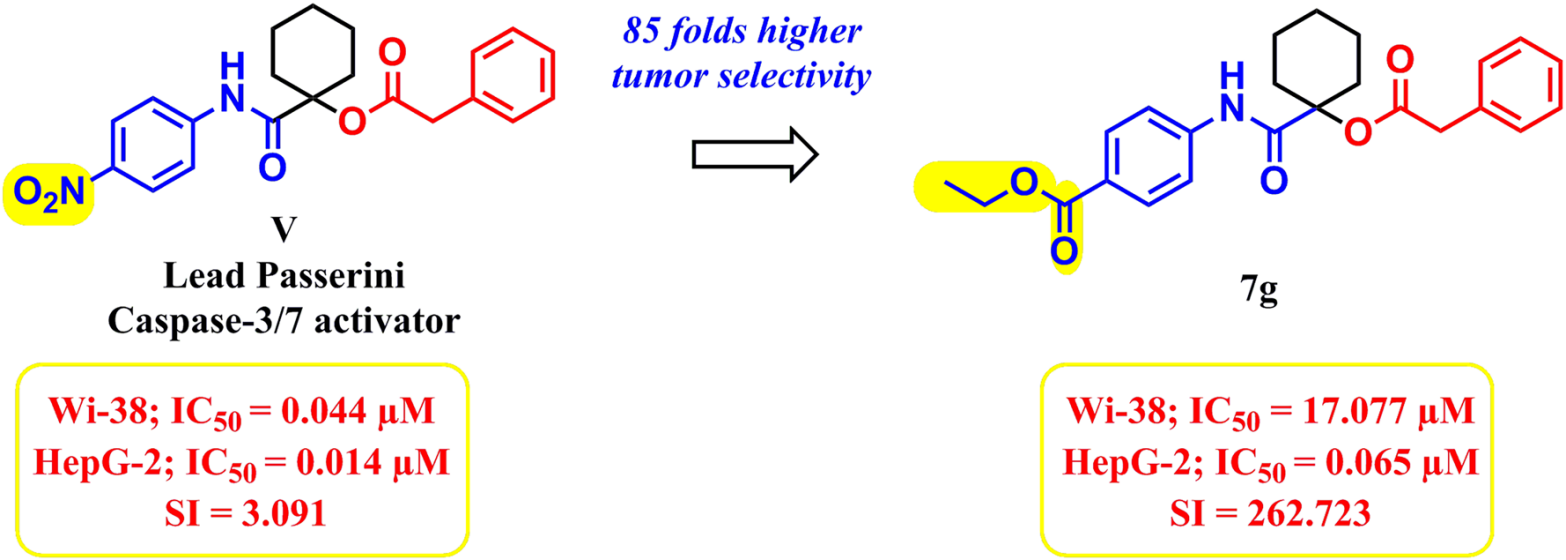
The effect of substituting nitro group by ester on the cytotoxicity profile of Passerini scaffold.

It is worth mentioning that the observed cytotoxicity pattern of the investigated Passerini esters highlighted their promising tumor selectivity profile. This could be safely deduced when compared with the cytotoxicity profiles of the previously studied Passerini leads with nitro group substituents^20^ as exemplified by the MTT assay data of the structurally related derivatives illustrated in Fig. 7. It is obvious that simple replacement of the nitro group by an ester moiety enhanced the tumor selectivity profile of the Passerini scaffold by approximately 85 folds reflecting their higher safety on normal cells.

Interestingly, this observation justified our design strategy and optimization protocol adopting replacement of the nitro group being considered as structural alert by the biocompatible ester group.

#### Apoptosis studies

##### Caspase-dependent apoptosis

Caspase-3/7 activation assay^20,42^ was performed to the most active anticancer derivatives to record the relative caspase-3/7 activity fold increments in HepG-2 and Caco-2 cells after treatment with IC_50_ doses (Table 1) of the studied compounds. Results (Table 2) showed that the three compounds activated caspase-3/7 and induced cancer cell apoptosis. Slightly higher activation potentials (3.08–4.18 folds) were detected in Caco-2 cells compared to HepG-2 (2.94–4.11 folds). The most potent antiproliferative Passerini adduct **7g** displayed the highest caspase-3/7 relative fold increments (up to 4.17) in the studied cancer cells compared to the other derivatives **7j**, **7a** and the reference drug, respectively. These results echoed the MTT assay results (Table 1). Thus, it could be postulated that caspase-3/7 mediated apoptotic induction may be the possible antiproliferative mechanism of the studied compounds.

**Table 2:** Relative fold increment in caspase-3/7 activity in Caco-2 and HepG-2 cells treated with IC_50_ doses of selected Passerini adducts.


Compound No.	R	Relative fold increment in caspase 3/7 activity	
		Caco-2	HepG-2
**7a**		3.086 ± 0.038	2.938 ± 0.063
**7g**		4.175 ± 0.189	4.118 ± 0.083
**7j**		3.586 ± 0.098	2.960 ± 0.065
**5-FU**	**–**	1.016 ± 0.009	1.001 ± 0.001

*All the values are expressed as mean ± SEM. 5-FU stands for 5-fluorouracil.

#### Bcl2 expression

Bcl2 protein inhibits apoptosis and is normally cleaved at Asp-34 during apoptosis by caspases or in vitro by recombinant caspase-3^29^. Furthermore, several studies demonstrated that Bcl2 expression is downregulated in various tissues as a result of caspase-3 activation^43^ and vice versa^44^ In this study, RT-qPCR of Bcl2 results showed that the studied compounds could repress Bcl2 expression by more than 10 folds relative to 5-FU-treated Caco-2 cells (Table 3). **7g** revealed the highest potential for downregulating Bcl2 by 11.5 folds, followed by **7j** and **7a.**

**Table 3:** Relative fold change in Bcl2 gene expression in Caco-2 cells treated with the selected Passerini adducts (0.06 µM for 72 h).

Compound No.	Bcl2 gene relative fold change
7a	0.471 ± 0.051
7g	0.087 ± 0.009
7j	0.127 ± 0.009
5-FU	0.948 ± 0.027

*All the values are expressed as mean ± SEM. 5-FU stands for 5-fluorouracil.

#### Morphological examination of the induced apoptosis

**Figure 8:**
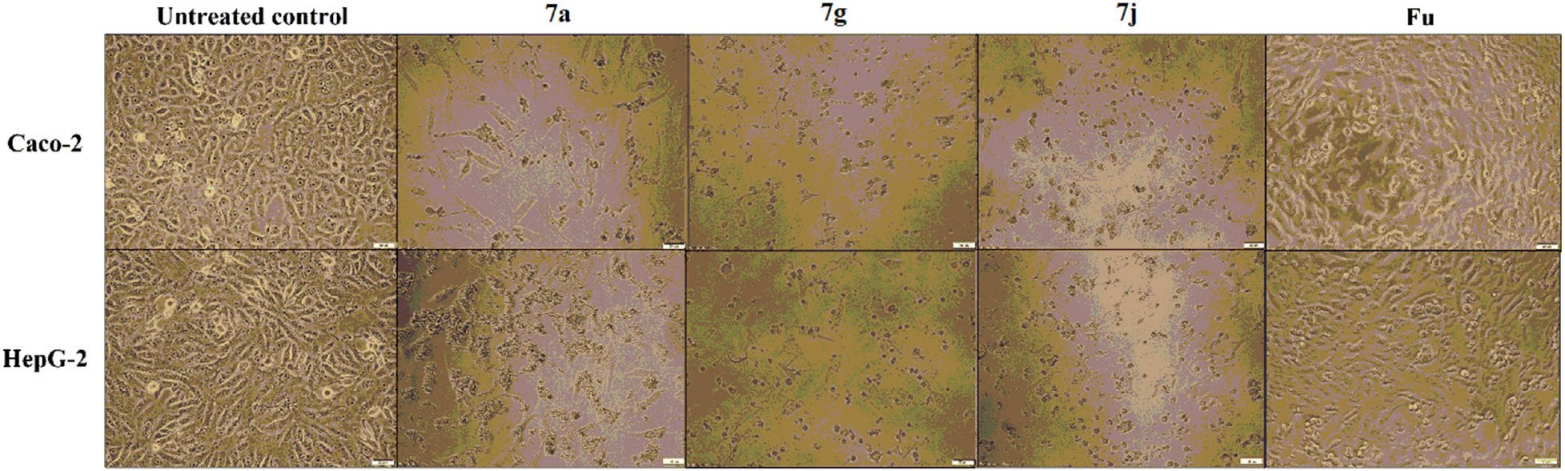
Morphological alterations of the hit compounds **7a**, **7g** and **7j**-treated cancer cell lines after 72 h, untreated cancer cells and 5-fluororuracil-treated cells.

Caco-2 and HepG-2 cells treated with the hit derivatives **7a, 7g** and **7j** were microscopically examined, after 72 h, compared to untreated and 5-FU-treated cells (Fig. 8). As shown, the hit compounds-treated cells appeared with severe shrinkage and morphological alterations indicating antiproliferative activities and high apoptotic induction capacities of the studied adducts.

#### Flow cytometric analysis of induced apoptosis

**Figure 9:**
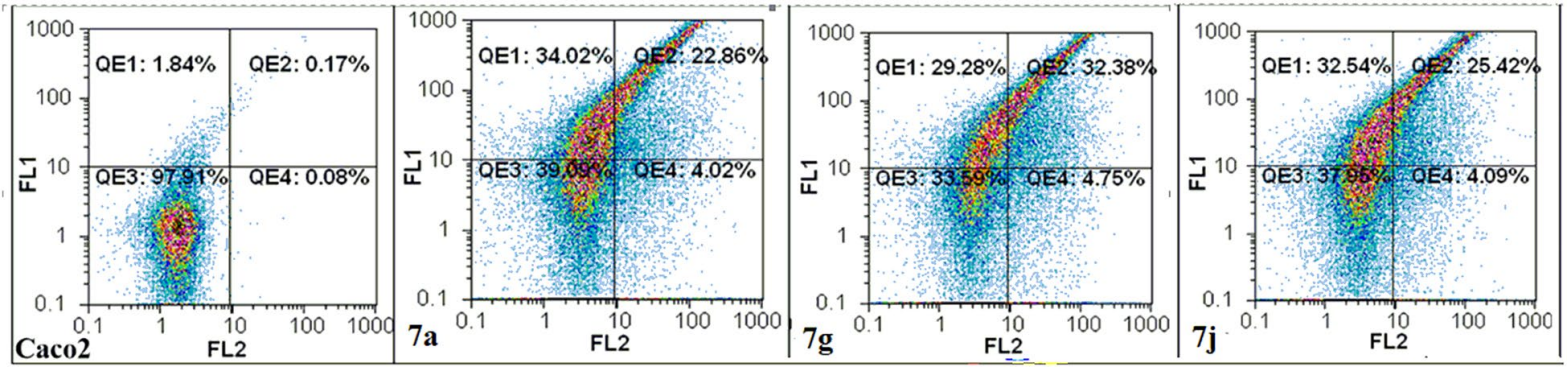
Flow cytometric analysis of apoptosis induction in the most active compounds-treated Caco-2 compared to untreated control cells, after staining with annexin V/propidium iodide.

Flow cytometric analysis was performed on the annexin-stained apoptotic Caco-2 cells treated with IC_50_ doses of the hit derivative **7a**, **7g** and **7j** for 72 h. Caco-2 cells were selected for the study being more sensitive to the caspase-dependent apoptotic potential of the studied compounds compared to HepG-2 cells. Herein, results showed that our efficient caspase activators induced cancer cells apoptosis by ≥ 51%. The most active caspase activator **7g** displayed the highest apoptotic induction potential as indicated by 58.7% total apoptotic cell population in the treated cells, followed by **7j (**52.6%) and **7a (**50.9%), respectively, compared to the untreated control (1.8%) as shown in Table 4 and Fig. 9. Interestingly, the detected potencies could be correlated to the results of MTT and caspase activation assays.

**Table 4:** Total apoptotic cell populations % in the hit compounds-treated Caco-2 cells.

Compound No.	% Apoptosis
Untreated control	1.815 ± 0.195
7a	50.905 ± 5.975
7g	58.730 ± 2.930
7j	52.650 ± 5.310

*All the values are demonstrated as mean ± SEM.

### Computational prediction of the hits’ ligand efficiency metrics, physicochemical properties, ADMET, and drug-likeness

As useful ligand identification tool, computational studies are usually performed to predict the most promising compounds ligand efficiency metrics, physicochemical and pharmacokinetic parameters, as well as drug-likeness. In the current study, the ligand efficiency (LE) and lipophilic ligand efficiency (LLE) metrics of the most promising caspase activators **7a**, **7g** and **7j** were computed (Table 5) as reported^20,45,46^. LE represents molecular size to potency balance^47–49^, whereas LLE illustrates how efficiently the active compound exploits its lipophilic character to exert potency^50^, accordingly, the hit compounds could be more reliably prioritized corrected for their molecular sizes and lipophilicity.

**Table 5:** Ligand efficiency (LE) and lipophilic ligand efficiency (LLE) metrics of selected Passerini adducts.

Compound No.	LogP^a^	NHA	Caco-2			HepG-2		
			pIC_50_	LE	LLE	pIC_50_	LE	LLE
**7a**	3.03	33	7.055	0.292	4.025	6.939	0.288	3.909
**7g**	3.74	30	7.193	0.328	3.453	7.187	0.328	3.447
**7j**	3.70	41	7.161	0.239	3.461	7.060	0.235	3.360

^a^LogP: logarithm of compound’s partition coefficient between *n*-octanol–water. ^b^NHA = non-hydrogen atom. ^c^pIC_50_: half-maximal inhibitory concentration (in term of molar concentration). pIC_50_ = -log (IC_50_). ^c^LE: ligand efficiency. LE = 1.37 (pIC_50_)/NHA. The lower acceptable limit of LE is 0.3^45^. ^d^LLE: lipophilic ligand efficiency. LLE = pIC_50_–cLog P. LLE values ≥ 3 are acceptable for lead compound, while values ≥ 5 are recommended for drug-like candidate^45,46^.

Results (Table 5) displayed promising LE and LLE metrics of the studied compounds. **7g** exceeded the acceptable LE limit against the screened cell lines. **7a** was slightly beyond the lower LE limit, whereas **7j** displayed the lowest predicted LE value among the group**.** Interestingly, the three compounds recorded lead-like LLE values ranging from 3.36 to 4.02.

Importantly, the physicochemical properties that formulate Lipinski's rule of five^51^, Veber and Egan`s rules^52,53^ bioavailability parameters were computed utilizing *SwissADME* web tool^54^ to assess the predicted drug-likeness of the studied compounds. Results (Table 6) showed that the three compounds were in full accordance with Egan’s parameters. **7a** and **7g** fulfilled Lipinski’s and Veber’s parameters, unlike **7j** that violated Lipinski’s molecular weight limit and Veber’s number of rotors. Topological polar surface area (TPSA) and aqueous solubility were also predicted being considered as useful descriptors of bioavailability^55^. The studied compounds recorded drug-like TPSA (150 < A^2^)^56,57^, acceptable solubility requirement, with **7g** at the top of the list**.**

**Table 6:** In silico prediction of physicochemical properties, ADMET and drug-likeness of selected Passerini adducts.

Parameter	7a	7g	7j
**Physicochemical properties**			
LogP	3.03	3.74	3.70
M.Wt	448.51	409.47	558.62
HBA	5	5	7
HBD	2	1	2
NROTB	10	10	15
TPSA	97.49	81.70	120.03
S	0.29	2.97	0.02
**ADMET**			
HIA	92.60	96.65	95.58
PPB	90.83	92.69	92.56
BBB	1.89	0.10	0.22
Caco2	32.73	30.08	25.18
MDCK	0.08	6.74	0.07
CYP2D6 inhibitor	Non	Non	Non
LD_50_	530	1739	800
Hepatoxic	Non	Non	Non
Carcinogenic	Non	Non	Non
Mutagenic	Non	Non	Non
**Drug-likeness**			
Lipinski	Yes	Yes	M.Wt ˃ 500
Veber	Yes	Yes	Rotors ˃ 10
Egan	Yes	Yes	Yes
Bioavailability score	0.55	0.55	0.55

*Pre-ADMET*^58^ and *ProTox-II*^59^ online servers were employed to predict the ADMET parameters for the current study hits. The three compounds recorded high predicted intestinal absorption (> 92.6%), medium Caco-2 (P_Caco2_ = 25.18–32.72 nm/s) and low MDCK (P_MDCK_ = 0.07–6.74 nm/s) models permeabilities, strong plasma proteins binding (90.83–92.69%) and medium CNS absorption (BBB = 0.10–1.89). The compounds were predicted to be devoid of CYP2D6 inhibition and were classified as class IV chemicals according to the Globally Harmonized System for Classification and Labeling of Classified Chemicals (GHS), with high predicted median lethal oral dose (LD_50_ = 530–1739 mg/kg) in rodents. Additional predicted toxicity assessment showed that the three studied compounds were not expected to be hepatotoxic, carcinogens nor mutagens, thus they could be predicted to be safe druggable leads.

The references values were considered as previously reported for TPSA^56,57^, HIA^60^, PPB^58^, BBB^61^, Caco2^62,63^, MDCK^64^, LD_50_^59^, Lipinski rule^51^, Veber’s rule^52^ and Egan’s rule^53^.

#### Structure–Activity relationship

The observed activity revealed that the currently modified Passerini scaffold generally conserved the intrinsic potency of the leads (Fig. 1), with enhanced tumor cell selectivity. However, both potency and selectivity were functions of the acyloxy counterpart nature and size. The bioinspired 2**-**(1*H*-indol-3-yl)acetate ester (**7a**) conferred excellent potency and selectivity to the main scaffold against screened cancer cell lines. Substituting 2**-**(1*H*-indol-3-yl)acetate motif was substituted with quinoline-2-carboxylate (**7b**) lowered the molecule’s potency and selectivity by nearly 2 folds. Structure simplification approach of the acyloxy counterpart of (**7a**) to benzoate (**7c**) attenuated the antiproliferative potency by 3 folds against Caco-2 cells and, by 1.5 folds against HepG-2 cells. This was mirrored by lowered selectivity to Caco-2 (5 folds) and HepG-2 (2 folds). Further substitution of the benzoate group with *o*-chloro (**7e**) or *o*-iodo (**7f**) led to increase in potency (2 folds) and selectivity (6 folds) against Caco-2 cells and supplemental enhancement in activity against HepG-2 with 2–3 folds more selectivity compared to the unsubstituted derivative (**7c).** Shifting the chloro substituent in **(7e)** to the *para-* position **(7d)** didn’t favor both potency and selectivity against the studied cell lines. The scaffold’s potency and selectivity profiles were optimized by modifying the benzoate group to phenylacetate (**7g**), or phenoxycarbonyl phenylalaninate (**7j**) that afforded the highest recorded potency and selectivity with the evaluated series. Other versatile substituents namely trifluoromethylcinnamate (**7h**), 4-(boc)aminobenzoylglycinate (**7i**) or thioglycolate (**7k**) exhibited relatively moderate activities being superior to the benzoate derivative (**7c**) against Caco-2 and comparable to it against HepG-2**.** Importantly, the hit antiproliferative derivatives recorded similar caspase-3/7 activation potential pattern highlighting the possibility that caspase-3/7 mediated apoptotic induction may be their main antiproliferative mechanism.

## Conclusion

This work portrays structure optimization of a promising Passerini-derived caspase activator scaffold towards enhanced tumor selectivity via excluding a possible toxicophore, introducing bioinspired substituents, and structure-simplification design approaches. Initial screening revealed excellent selectivity against colorectal Caco-2 and hepatocellular HepG-2 cancer cells rather than normal ones (SI up to 266.8). All derivatives were superior to 5-fluorouracil with sub-micromolar IC_50_ values. **7a, 7g** and **7j** were the study hits. The three derivatives activated caspase up to 4.2 folds in the treated cancer cells, downregulated Bcl2 and induced apoptosis (up to 58.7%). Computational studies predicted their acceptable efficiency metrics and drug-like pharmacokinetic parameters.

## Experimental

### Chemistry

The Materials and equipment were described in the supporting information.

#### Synthesis of 4-amino ethyl benzoate (2)

A solution of 4-amino benzoic acid **1** (20 g, 0.14 mol) in anhydrous EtOH (150 ml) and sulphuric acid (17 ml) was heated until the consumption of the acid was complete (typically 1–2 h). After cooling, the mixture was neutralized by using solution of washing soda. The formed desired product was filtered, and washed using distilled water to yield a white solid of **2** (22 g, 92%); m.p = 74–76 °C.

#### Synthesis of 4-Formamido ethyl benzoate 3^35^.

To a solution of ethyl 4-aminobenzoate **2** (11 g, 0.06 mol) in toluene (100 ml), formic acid (3.98 g, 0.08 mol) was added dropwise, followed by heating to 85–90 °C for 2 h. After cooling at r.t, the product was formed, filtered and washed using distilled water to yield a white solid of **3** (10.2 g, 79%); m.p = 138–140 °C.

#### Synthesis of ethyl-4-isocyanobenzoate 4^36^

To a mixture of formamide **3** (5.0 g, 0.026 mol), I_2_ (9.87 g, 0.039 mol) and Ph_3_P (10 g, 0.038 mol) in DCM (75 ml), Et_3_N (7.79 g, 10.7 ml, 0.07 mol) was added dropwise. The reaction mixture was stirred at r.t for 1 h. The reaction mixture was diluted by adding CHCl_3_ (50 ml) and then washed using an ice-cold saturated solution of Na_2_S_2_O_3_. The aqueous phase was extracted using DCM and the organic phase was washed with brine, dried over anhydrous Na_2_SO_4_, and filtered. The combined organic phase was evaporated and purified using column chromatography (1:2, EtOAc–*n*-hexane) to give the corresponding brown solid of isocyanide **4** (2 g, 55%); m.p = 93–96 °C, lit.^36^ 101–105 °C.

#### General method for Passerini reactions^20^

A mixture of ethyl-4-isocyanobenzoate **4** (50 mg, 0.286 mmol, 0.5 eq), carboxylic acids derivatives namely indole-3-acetic acid, quinaldic acid, benzoic acid, 4-chlorobenzoic acid, 2-chlorobenzoic acid, 2-iodobenzoic acid, phenylacetic acid, *(E)-*3-(4-(trifluoromethyl)cinnamic acid, *N*-Boc-4-aminohippuric acid, (phenoxycarbonyl)-l-phenylalanine, and 2-mercaptoacetic acid **5a–k,** respectively (0.57 mmol, 1 eq) and excess cyclohexanone **6** (2.86 mmol, 5 eq) was stirred at r.t for 24 h and was monitored by TLC. Then, the reaction was diluted using 5 ml of DCM and neutralized using saturated sodium bicarbonate solution. The organic layer was collected, dried using anhydrous Na_2_SO_4_ and purified using flash column chromatography (1:3, EtOAc–*n*-hexane).

##### Ethyl-4-(1-(2-(1*H*-indol-3-yl)acetoxy)cyclohexanecarboxamido)benzoate 7a

Orange powder; yield 50%; m.p = 79–81 °C; R_f_ 0.25 (1:2, EtOAc–*n*-hexane); IR: *v*_max_/cm^−1^ 3372 (OC**NH**), 1697 (br, OC, NCO); ^**1**^**H-NMR** (500 MHz, DMSO-*d*_6_) δ_H_: 10.94 (s, 1H, **NH**-CH), 9.88 (s, 1H, OC-**NH**), 7.88 (d, *J* = 9.0 Hz, 2H, Ar–**H**), 7.77 (d, *J* = 9.0 Hz, 2H, Ar–**H**), 7.50 (d, *J* = 8.0 Hz, 1H, Ar–**H**), 7.33 (d, *J* = 8.5 Hz, 1H, Ar–**H**), 7.25 (d, *J* = 2.0 Hz, 1H, Ar–**H**), 7.04 (t, *J* = 7.5 Hz, 1H, Ar–**H**), 6.91 (t, *J* = 7.0 Hz, 1H, Ar–**H**), 4.27 (q, *J* = 7.5 Hz, 2H, OC**H**_**2**_–CH_3_), 3.87 (s, 2H, OC–**CH**_**2**_), 2.13 (d, *J* = 13.5 Hz, 2H, *c*-Hex-**H**), 1.77–1.71 (td, *J* = 14.5, 4.0 Hz, 2H, *c*-Hex-**H**), 1.59–1.50 (m, 3H, *c*-Hex-**H**), 1.41–1.34 (m, 2H, *c*-Hex-**H**), 1.30 (t, *J* = 7.5 Hz, 3H, OCH_2_–C**H**_**3**_), 1.25–1.20 (m, 1H, *c*-Hex-**H**); ^**13**^**C-NMR** (125 MHz, DMSO-*d*_6_) δ_C_: 171.6 (O**C**NH), 170.6 (C–O–**CO**), 165.4 (EtO–**CO**), 143.4, 136.1, 129.9, 127.1, 124.3, 124.2, 121.1, 119.4, 118.5, 118.4, 111.4, 106.9 (Ar–**C**), 81.2 (O–**C**–CO), 60.5 (O**C**H_2_), 31.3, 31.0, 24.6, 20.8 (*c*-Hex-**C**), 14.3 (**C**H_3_). Anal. calcd. for C_26_H_28_N_2_O_5_ (448.51): C, 69.63; H, 6.29; N, 6.25; found C, 69.42; H, 6.18; N, 6.43.

##### 1-((4-(Ethoxycarbonyl)phenyl)carbamoyl)cyclohexyl quinoline-2-carboxylate 7b

Orange powder; yield 56%; m.p = 103–105 °C; R_f_ 0.32 (1:2, EtOAc–*n*-hexane); IR: *v*_max_/cm^–1^ 3398 (OC**NH**), 1717, 1680 (OC, NCO); ^**1**^**H-NMR** (500 MHz, DMSO-*d*_6_) δ_H_: 10.07 (s, 1H, –N**H**), 8.58 (d, *J* = 9.0 Hz, 1H, Ar–**H**), 8.17 (d, *J* = 8.5 Hz, 1H, Ar–**H**), 8.13 (d, *J* = 8.5 Hz, 1H, Ar–**H**), 8.08 (d, *J* = 8.0 Hz, 1H, Ar–**H**), 7.87–7.83 (m, 3H, Ar–**H**), 7.76–7.71 (m, 3H, Ar–**H**), 4.23 (q, *J* = 6.5 Hz, 2H, OC**H**_**2**_–CH_3_), 2.33 (d, *J* = 14.0 Hz, 2H, *c*-Hex-**H**), 2.04 (s, 1H, *c*-Hex-**H**), 1.95–1.89 (m, 2H, *c*-Hex-**H**), 1.66 (s, 5H, *c*-Hex-**H**), 1.25 (t, *J* = 7.5 Hz, 3H, OCH_2_–C**H**_**3**_); ^**13**^**C-NMR** (125 MHz, DMSO-*d*_6_) δ_C_: 171.1 (O**C**NH), 165.4 (EtO–**C**O), 163.2 (C–O–**CO**), 147.8, 146.9, 143.4, 137.8, 130.8, 129.9, 129.0, 128.9, 128.1, 124.4, 121.2, 119.6 (Ar–**C**), 82.7 (O–**C**–CO), 60.5 (O**C**H_2_), 31.5, 30.7, 24.6, 21.1 (*c*-Hex-**C**), 14.2 (**C**H_3_). Anal. calcd. for C_26_H_26_N_2_O_5_ (446.50): C, 69.94; H, 5.87; N, 6.27; found C, 70.01; H, 5.97; N, 6.32.

##### Ethyl-4-(1-(benzoyloxy)cyclohexanecarboxamido)benzoate 7c

Off-white powder; yield 55%; m.p = 140 – 142 °C; R_f_ 0.48 (1:2, EtOAc–*n*-hexane); IR: *v*_max_/cm^−1^ 3342 (OC**NH**), 1718, 1679 (OC, NCO); ^**1**^**H-NMR** (500 MHz, DMSO-*d*_6_) δ_H_: 9.96 (s, 1H, -N**H**), 8.04 (d, *J* = 7.5 Hz, 2H, Ar–**H**), 7.87 (d, *J* = 9.0 Hz, 2H, Ar–**H**), 7.76 (d, *J* = 9.0 Hz, 2H, Ar–**H**), 7.69 (t, *J* = 7.0 Hz, 1H, Ar–**H**), 7.56 (t, *J* = 7.5 Hz, 2H, Ar–**H**), 4.26 (q, *J* = 7.0 Hz, 2H, OC**H**_**2**_–CH_3_), 2.31 (d, *J* = 13.5 Hz, 2H, *c*-Hex-**H**), 1.88 (td, *J* = 14.0, 2.5 Hz, 2H, *c*-Hex-**H**), 1.67 (d, *J* = 10.5 Hz, 3H, *c*-Hex-**H**), 1.60–1.51 (m, 2H, *c*-Hex-**H**), 1.33–1.31 (m, 1H, *c*-Hex-**H**), 1.28 (t, *J* = 7.5 Hz, 3H, OCH_2_–C**H**_**3**_); ^**13**^**C-NMR** (125 MHz, DMSO-*d*_6_) δ_C_: 171.3 (O**C**NH), 165.4 (EtO–**CO**), 164.4 (C–O–**CO**), 143.4, 133.6, 129.9, 129.6, 128.8, 124.4, 119.6 (Ar–**C**), 81.7 (O–**C**–CO), 60.5 (O**C**H_2_), 31.4, 24.6, 21.1 (*c*-Hex-**C**), 14.2 (**C**H_3_). Anal. calcd. for C_23_H_25_NO_5_ (395.45): C, 69.86; H, 6.37; N, 3.54; found C, 69.93; H, 6.42; N, 3.61.

##### 1-((4-(Ethoxycarbonyl)phenyl)carbamoyl)cyclohexyl 4-chlorobenzoate 7d

Grey powder; yield 56%; m.p = 193–195 °C; R_f_ 0.54 (1:2, EtOAc–*n*-hexane); IR: *v*_max_/cm^–1^ 3277 (OC**NH**), 1717, 1684 (OC, NCO); ^**1**^**H-NMR** (500 MHz, DMSO-*d*_6_) δ_H_: 9.92 (s, 1H, –N**H**), 8.00 (d, *J* = 9.0 Hz, 2H, Ar–**H**), 7.84 (d, *J* = 8.5 Hz, 2H, Ar–**H**), 7.71 (d, *J* = 8.5 Hz, 2H, Ar–**H**), 7.60 (d, *J* = 8.5 Hz, 2H, Ar–**H**), 4.23 (q, *J* = 7.5 Hz, 2H, OC**H**_**2**_–CH_3_), 2.26 (d, *J* = 13.5 Hz, 2H, *c*-Hex-**H**), 1.85 (td, *J* = 13.5, 2.5 Hz, 2H, *c*-Hex-**H**), 1.63 (d, *J* = 9.5 Hz, 3H, *c*-Hex-**H**), 1.55–1.48 (m, 2H, *c*-Hex-**H**), 1.32–1.28 (m, 1H, *c*-Hex-**H**), 1.25 (t, *J* = 7.0 Hz, 3H, OCH_2_–C**H**_**3**_); ^**13**^**C-NMR** (125 MHz, DMSO-*d*_6_) δ_C_: 171.1 (O**C**NH), 165.3 (EtO–**CO**), 163.6 (C–O–**CO**), 143.3, 138.5, 131.4, 130.0, 129.0, 128.7, 124.4, 119.6 (Ar–**C**), 82.1 (O–**C**–CO), 60.5 (O**C**H_2_), 31.4, 30.7, 24.5, 21.1 (*c*-Hex-**C**), 14.2 (**C**H_3_). Anal. calcd. for C_23_H_24_ClNO_5_ (429.89): C, 64.26; H, 5.63; N, 3.26; found C, 64.19; H, 5.53; N, 3.39.

##### 1-((4-(Ethoxycarbonyl)phenyl)carbamoyl)cyclohexyl 2-chlorobenzoate 7e

White powder; yield 51%; m.p = 130–132 °C; R_f_ 0.42 (1:2, EtOAc–*n*-hexane); IR: *v*_max_/cm^−1^ 3341 (OC**NH**), 1729, 1692 (OC, NCO); ^**1**^**H-NMR** (500 MHz, DMSO-*d*_6_) δ_H_: 10.02 (s, 1H, −N**H**), 7.94–7.92 (m, 1H, Ar–**H**), 7.89 (d, *J* = 8.5 Hz, 2H, Ar–**H**), 7.78 (d, *J* = 9.0 Hz, 2H, Ar–**H**), 7.63–7.59 (m, 2H, Ar–**H**), 7.53–7.50 (m, 1H, Ar–**H**), 4.27 (q, *J* = 14.0, 7.0 Hz, 2H, OC**H**_**2**_–CH_3_), 2.31 (d, *J* = 13.5 Hz, 2H, *c*-Hex-**H**), 1.94–1.90 (td, *J* = 13.5, 3.0 Hz, 2H, *c*-Hex-**H**), 1.68–1.61 (m, 5H, *c*-Hex-**H**), 1.34–1.32 (m, 1H, *c*-Hex-**H**), 1.29 (t, *J* = 7.0 Hz, 3H, OCH_2_–C**H**_**3**_); ^**13**^**C-NMR** (125 MHz, DMSO-*d*_6_) δ_C_: 170.9 (O**C**NH), 165.4 (EtO–**CO**), 163.8 (C–O–**CO**), 143.4, 133.5, 132.0, 131.4, 130.9, 130.2, 130.0, 127.5, 124.5, 119.6 (Ar–**C**), 83.1 (O–**C**–CO), 60.5 (O**C**H_2_), 31.3, 24.5, 21.1 (*c*-Hex-**C**), 14.3 (**C**H_3_). Anal. calcd. for C_23_H_24_ClNO_5_ (429.89): C, 64.26; H, 5.63; N, 3.26; found C, 64.33; H, 5.71; N, 3.42.

##### 1-((4-(Ethoxycarbonyl)phenyl)carbamoyl)cyclohexyl 2-iodobenzoate 7f

Off-white powder; yield 76%; m.p = 148–150 °C; R_f_ 0.45 (1:2, EtOAc–*n*-hexane); IR: *v*_max_/cm^−1^ 3328 (OC**NH**), 1730, 1693 (OC, NCO); ^**1**^**H-NMR** (500 MHz, DMSO-*d*_6_) δ_H_: 10.00 (s, 1H, –N**H**), 8.02 (d, *J* = 7.0 Hz, 1H, Ar–**H**), 7.93 (dd, *J* = 7.5, 2.0 Hz, 1H, Ar–**H**), 7.88 (d, *J* = 8.5 Hz, 2H, Ar–**H**), 7.79 (d, *J* = 9.0 Hz, 2H, Ar–**H**), 7.57 (t, *J* = 6.5 Hz, 1H, Ar–**H**), 7.30 (td, *J* = 8.0, 1.5 Hz, 1H, Ar–**H**), 4.26 (q, *J* = 7.5 Hz, 2H, OC**H**_**2**_–CH_3_), 2.31 (d, *J* = 14.0 Hz, 2H, *c*-Hex-**H**), 1.91 (td, *J* = 13.5, 3.0 Hz, 2H, *c*-Hex-**H**), 1.68–1.61 (m, 5H, *c*-Hex-**H**), 1.38–1.32 (m, 1H, *c*-Hex-**H**), 1.29 (t, *J* = 7.5 Hz, 3H, OCH_2_–C**H**_**3**_); ^**13**^**C-NMR** (125 MHz, DMSO-*d*_6_) δ_C_: 170.9 (O**C**NH), 165.4 (EtO–**CO**), 165.2 (C–O–**CO**), 143.4, 140.9, 135.4, 133.3, 130.8, 130.0, 128.3, 124.4, 119.7 (Ar–**C**), 94.7 (**C**-I), 82.9 (O–**C**–CO), 60.5 (O**C**H_2_), 31.3, 24.5, 21.1 (*c*-Hex-**C**), 14.3 (**C**H_3_). Anal. calcd. for C_23_H_24_INO_5_ (521.34): C, 52.99; H, 4.64; N, 2.69; found C, 53.11; H, 4.88; N, 2.91.

##### Ethyl -4-(1-(2-phenylacetoxy)cyclohexanecarboxamido)benzoate 7g

Off-white powder; yield 72%; m.p = 90 – 93 °C; R_f_ 0.48 (1:2, EtOAc–*n*-hexane); IR: *v*_max_/cm^−1^ 3386 (OC**NH**), 1737, 1708, 1681 (OC, NCO); ^**1**^**H-NMR** (500 MHz, DMSO-*d*_6_) δ_H_: 9.87 (s, 1H, –N**H**), 7.89 (d, *J* = 9.0 Hz, 2H, Ar–**H**), 7.77 (d, *J* = 9.0 Hz, 2H, Ar–**H**), 7.33–7.28 (m, 4H, Ar–**H**), 7.26–7.23 (m, 1H, Ar–**H**), 4.27 (q, *J* = 7.0 Hz, 2H, O**CH**_**2**_–CH_3_), 3.78 (s, 2H, CO–**CH**_**2**_–Ar), 2.12 (d, *J* = 13.5 Hz, 2H, *c*-Hex-**H**), 1.74 (td, *J* = 14.0, 3.5 Hz, 2H, *c*-Hex-**H**), 1.53–1.50 (m, 3H, *c*-Hex-**H**), 1.35–1.32 (m, 2H, *c*-Hex-**H**), 1.29 (t, *J* = 7.5 Hz, 3H, OCH_2_–C**H**_**3**_), 1.24–1.19 (m, 1H, *c*-Hex-**H**); ^**13**^**C-NMR** (125 MHz, DMSO-*d*_6_) δ_C_: 171.3 (O**C**NH), 170.1 (C–O–**CO**), 165.4 (EtO–**CO**), 143.3, 134.3, 129.9, 129.5, 128.4, 128.3, 126.9, 124.4, 119.5 (Ar–**C**), 81.4 (O–**C**–CO), 60.5 (O**C**H_2_), 40.9 (CO–**CH**_**2**_), 31.3, 24.5, 20.7 (*c*-Hex-**C**), 14.2 (**C**H_3_). Anal. calcd. for C_24_H_27_NO_5_ (409.47): C, 70.40; H, 6.65; N, 3.42; found C, 70.66; H, 6.81; N, 3.70.

##### Ethyl-4-(1-((3-(4-(trifluoromethyl)phenyl)acryloyl)oxy)cyclohexanecarboxamido)benzoate 7 h

Orange crystal; yield 57%; m.p = 145–147 °C; R_f_ 0.80 (1:2, EtOAc–*n*-hexane); IR: *v*_max_/cm^–1^ 3343 (OC**NH**), 1715, 1639 (OC, NCO); ^**1**^**H-NMR** (500 MHz, DMSO-*d*_6_) δ_H_: 9.93 (s, 1H, –N**H**), 7.95 (d, *J* = 8.0 Hz, 2H, Ar–**H**), 7.84 (d, *J* = 9.0 Hz, 2H, Ar–**H**), 7.76–7.73 (m, 4H, Ar–**H**), 7.71 (s, 1H, Ar–C**H**=CH), 6.86 (d, *J* = 16.0 Hz, 1H, Ar–CH=C**H**), 4.23 (q, *J* = 7.0 Hz, 2H, OC**H**_**2**_–CH_3_), 2.20 (d, *J* = 14.0 Hz, 2H, *c*-Hex-**H**), 1.81 (td, *J* = 16.0 ,4.0 Hz, 2H, *c*-Hex-**H**), 1.62–1.45 (m, 6H, *c*-Hex-**H**), 1.26 (t, *J* = 7.5 Hz, 3H, OCH_2_–C**H**_**3**_); ^**13**^**C-NMR** (125 MHz, DMSO-*d*_6_) δ_C_: 171.8 (O**C**NH), 165.9 (EtO–**CO**), 165.1 (C–O–**CO**), 143.9 (CH=**C**H–Ar), 143.6, 138.5, 130.4, 129.6, 126.3, 124.8 (**C**F_3_), 121.7 (Ar–**C**), 120.0 (CH=**C**H–Ar), 82.0 (O–**C**–CO), 61.0 (O**C**H_2_), 31.9, 25.1, 21.5 (*c*-Hex-**C**), 14.7 (**C**H_3_). Anal. calcd. for C_26_H_26_F_3_NO_5_ (489.48): C, 63.80; H, 5.35; N, 2.86; found C, 63.99; H, 5.52; N, 3.08.

##### Ethyl-4-(1-(2-(4-((tert-butoxycarbonyl)amino)benzamido)acetoxy)cyclohexane carboxamido) benzoate 7i

Reddish brown powder; yield 62%; m.p = 115–117 °C; R_f_ 0.26 (1:2, EtOAc–*n*-hexane); IR: *v*_max_/cm^−1^ 3334 (br, NH's), 1713, 1644 (OC, NCO); ^**1**^**H-NMR** (500 MHz, DMSO-*d*_*6*_) δ_H_: 9.74, 9.62 (2 s, 2H, 2NH's), 8.90 (t, *J* = 5.5 Hz, 1H, CH_2_–**NH**), 7.88–7.71 (m, 6H, Ar–**H**), 7.48 (d, *J* = 9.0 Hz, 2H, Ar–**H**), 4.26–4.22 (m, 3H, **CH**_**2**_–NH, OC**H**_**2**_–CH_3_), 4.08 (d, *J* = 5.5 Hz, 1H, **CH**_**2**_–NH), 2.11 (d, *J* = 12.5 Hz, 2H, *c*-Hex-**H**), 2.04 (s, 2H, *c*-Hex-**H**), 1.75 (td, *J* = 14.5, 4.5 Hz, 2H, *c*-Hex-**H**), 1.55 (bs, 4H, *c*-Hex-**H**), 1.44 (s, 9H, C(**CH**_**3**_)_3_), 1.26 (t, *J* = 7.0 Hz, 3H, OCH_2_–C**H**_**3**_). ^**13**^**C-NMR** (125 MHz, DMSO-*d*_*6*_) δ_C_: 171.2 (O**C**NH), 169.0 (EtO–**CO**), 166.7 (CH_2_–NH–**CO**), 165.4 (C–O–**CO**), 152.6 (NH–**CO**–O), 143.4, 143.2, 142.7, 130.0, 129.9, 128.2, 126.7, 124.4, 119.5, 117.2 (Ar–**C**), 81.9 (O–**C**–CO), 79.6 (**C**(CH_3_)_3_), 60.5 (O**C**H_2_), 41.8 (**CH**_**2**_-NH), 31.5, 30.7, 28.1 (C(**CH**_**3**_)_3_), 20.7 (*c*-Hex-**C**), 14.2 (C**H**_**3**_). Anal. calcd. for C_30_H_37_N_3_O_8_ (567.63): C, 63.48; H, 6.57; N, 7.40; found C, 63.66; H, 6.77; N, 7.88.

##### Ethyl-4-(1-((2-((phenoxycarbonyl)amino)-3-phenylpropanoyl)oxy) cyclohexanecarboxamido) benzoate 7j

Reddish brown crystal; yield 71%; m.p = 103–105 °C; R_f_ 0.48 (1:2, EtOAc–*n*-hexane); IR: *v*_max_/cm^−1^ 3336 (br, NH's), 1709 (br, OC, NCO); ^**1**^**H-NMR** (500 MHz, DMSO-*d*_6_) δ_H_: 9.53 (s, 1H, –N**H**), 7.90 (d, *J* = 8.0 Hz, 1H, –N**H**), 7.86 (d, *J* = 8.5 Hz, 2H, Ar–**H**), 7.73 (d, *J* = 9.0 Hz, 2H, Ar–**H**), 7.28–7.17 (m, 10H, Ar–**H**), 4.95 (q, *J* = 12.5 Hz, 2H, Ar–C**H**_**2**_), 4.44–4.39 (m, 1H, C**H**), 4.24 (q, *J* = 7.0 Hz, 2H, OC**H**_**2**_–CH_3_), 3.15 (dd, *J* = 14.5, 5.0 Hz, 1H, *c*-Hex-**H**), 2.86 (dd, *J* = 13.5, 4.0 Hz, 1H, *c*-Hex-**H**), 2.08 (d, *J* = 11.0 Hz, 2H, *c*-Hex-**H**), 1.75–1.70 (m, 2H, *c*-Hex-**H**), 1.51 (d, *J* = 8.5 Hz, 3H, *c*-Hex-**H**), 1.43–1.34 (m, 1H, *c*-Hex-**H**), 1.26 (t, *J* = 7.5 Hz, 3H, OCH_2_–C**H**_**3**_); ^**13**^**C-NMR** (125 MHz, DMSO-*d*_6_) δ_C_: 171.0 (O–**CO**–CH), 170.6 (NH–**CO**–C), 165.4 (EtO–**CO**), 156.4 (NH–**O**–O), 143.1, 137.5, 136.8, 130.0, 129.1, 128.3, 128.2, 127.9, 127.6, 126.5, 124.5, 119.4 (Ar–**C**), 82.1 (O–**C**–CO), 65.6 (O**C**H_2_), 60.5 (**CH**–CH_2_), 55.6 (CH–**CH**_**2**_), 36.0, 31.4, 31.1, 24.5, 20.7 (*c*-Hex-**C**), 14.2 (**C**H_3_). Anal. calcd. for C_32_H_34_N_2_O_7_ (558.62): C, 68.80; H, 6.13; N, 5.01; found C, 69.11; H, 6.40; N, 5.33.

##### Ethyl-4-(1-(2-mercaptoacetoxy)cyclohexane-1-carboxamido)benzoate 7k

Yellow crystal; yield 50%; m.p = 108–110 °C; R_f_ 0.35 (1:2, EtOAc–*n*-hexane); IR: *v*_max_/cm^−1^ 3318 (OC**NH**), 2570 (SH), 1741, 1705, 1679 (OC, NCO); ^**1**^**H-NMR** (500 MHz, DMSO-*d*_6_) δ_H_: 9.84 (s, 1H, –N**H**), 7.87 (d, *J* = 8.5 Hz, 2H, Ar–**H**), 7.75 (d, *J* = 8.0 Hz, 2H, Ar–**H**), 4.26 (q, *J* = 7.0 Hz, 2H, OC**H**_**2**_–CH_3_), 4.03–3.80 (m, 2H, C**H**_**2**_-SH), 2.15 (d, *J* = 12.5 Hz, 2H, *c*-Hex-**H**), 1.79 (t, *J* = 12.0 Hz, 2H, *c*-Hex-**H**), 1.60–1.38 (m, 5H, *c*-Hex-**H**), 1.29 (t, *J* = 7.0 Hz, 3H, OCH_2_–C**H**_**3**_), 1.25–1.21 (m, 1H, *c*-Hex-**H**), 0.85–0.80 (m, 1H, –**SH**). ^**13**^**C-NMR** (125 MHz, DMSO-*d*_6_) δ_C_: 171.0 (O**C**NH), 168.1 (EtO–**CO**), 165.3 (O–**CO**–CH_2_), 143.2, 129.9, 124.5, 119.6, 118.6 (Ar–**C**), 82.4 (O–**C**–CO), 60.5 (O**C**H_2_), 40.7 (**C**H_2_-SH), 31.3, 24.5, 20.8 (*c*-Hex-**C**), 14.2 (**C**H_3_). Anal. calcd. for C_18_H_23_NO_5_S (365.44): C, 59.16; H, 6.34; N, 3.83; found C, 59.31; H, 6.51; N, 3.97.

##### Ethyl 4-(1-hydroxycyclohexanecarboxamido)benzoate 9

Reddish brown crystal; yield 50%; m.p = 160–163 °C; R_f_ 0.32 (1:2, EtOAc–*n*-hexane); IR: *v*_max_/cm^−1^ 3326 (OH), 3264 (OC**NH**), 1718, 1656 (OC, NCO); ^**1**^**H-NMR** (500 MHz, DMSO-*d*_6_) δ_H_: 9.91 (s, 1H, –N**H**), 7.85 (q, *J* = 12.0, 8.5 Hz, 4H, Ar–**H**), 5.49 (s, 1H, O**H**), 4.23 (q, *J* = 6.5 Hz, 2H, OC**H**_**2**_–CH_3_), 1.70 (td, *J* = 13.0, 4.0 Hz, 2H, *c*-Hex-**H**), 1.56 (d, *J* = 12.0 Hz, 5H, *c*-Hex-**H**), 1.52–1.47 (m, 2H, *c*-Hex-**H**), 1.26 (t, *J* = 7.0 Hz, 3H, OCH_2_–C**H**_**3**_), 1.19–1.14 (m, 1H, *c*-Hex-**H**); ^**13**^**C-NMR** (125 MHz, DMSO-*d*_6_) δ_C_: 177.1 (O**C**NH), 165.9 (EtO–**CO**), 143.7, 130.5, 124.8, 119.5 (Ar–C), 74.5 (O–**C**–CO), 60.9 (O**C**H_2_), 34.2, 31.2, 25.5, 21.3 (*c*-Hex-**C**), 14.7 (**C**H_3_). Anal. calcd. for C_16_H_21_NO_4_ (291.34): C, 65.96; H, 7.27; N, 4.81; found C, 66.12; H, 7.51; N, 4.90.

##### Ethyl-4-(2,4-dioxo-1-oxa-3-azaspiro[4.5]decan-3-yl)benzoate 11

Orange crystal; yield 60%; m.p = 143–145 °C; R_f_ 0.77 (1:2, EtOAc–*n*-hexane); IR: *v*_max_/cm^−1^ 1815 (CO–N–CO), 1736 (EtO–CO); ^**1**^**H-NMR** (500 MHz, DMSO-*d*_6_) δ_H_: 8.06 (d, *J* = 9.0 Hz, 2H, Ar–**H**), 7.62 (d, *J* = 8.5 Hz, 2H, Ar–**H**), 4.30 (q, *J* = 7.0 Hz, 2H, OC**H**_**2**_–CH_3_), 2.04 (s, 2H, *c*-Hex-**H**), 1.80 (td, *J* = 12.0, 4.0 Hz, 2H, *c*-Hex-**H**), 1.72–1.68 (m, 2H, *c*-Hex-**H**), 1.62–1.48 (m, 4H, *c*-Hex-**H**), 1.29 (t, *J* = 7.5 Hz, 3H, OCH_2_–C**H**_**3**_); ^**13**^**C-NMR** (125 MHz, DMSO-*d*_6_) δ_C_: 174.0 (N–**CO**), 165.0 (EtO–**CO**), 152.8 (N–**CO**–O), 135.4, 129.8, 129.7, 126.6 (Ar–**C**), 85.0 (O–**C**–CO), 61.1 (O**C**H_2_), 31.2, 30.7, 23.8, 20.8 (*c*-Hex-**C**), 14.1 (**C**H_3_). Anal. calcd. for C_17_H_19_NO_5_ (317.34): C, 64.34; H, 6.03; N, 4.41; found C, 64.64; H, 6.32; N, 4.52.

### Biological evaluation

#### Cytotoxicity screening

Wi-38, Caco-2 and HepG-2 cells were cultured in DMEM medium-contained 10% FBS, seeded as 5 × 10^3^ cells per well in 96-well cell culture plate and incubated at 37 °C in 5% CO_2_ incubator. After 24 h, serial concentrations of the test compounds were incubated with cells for 72 h. Cell viability was assayed via MTT protocol^27^ (Supplementary data). IC_50_ values of the synthesized compounds were measured via the Graphpad Instat software. Morphological changes were examined using phase contrast inverted microscope with a digital camera (Olympus, Japan).

#### Caspase-3/7 activation assay

ApoONE^®^ Caspase-3/7 kit was used following the manufacturer’s instructions (Supplementary data).

#### Real-time quantitative PCR Analysis of Bcl2

Colon cancer cell line (Caco-2) was incubated with the most effective anticancer compounds, at 0.06 µM, for 72 h in 5% CO_2_ incubator. RNAs of untreated and treated cancer cells were extracted using Gene JET RNA purification kit (Thermo Scientific, USA). Then cDNAs were synthesized using cDNA Synthesis Kit (Thermo Scientific, USA). Real time PCR was performed using SYBR green master mix and specific primers (Forward/Reverse) as detailed in the supplementary data.

#### Flow cytometric analysis of apoptosis

The hit compounds were assayed for their proapoptotic effects by incubation MDA-MB 231 cells for 72 h, at their minimum IC_50_ doses. After trypsinization, the treated and untreated cancer cells were stained with fluorescein isothiocyanate (FITC)-annexin V/ propidium iodide (PI) then subjected to flow cytometric analysis of apoptosis as previously reported^20^ (Supplementary data).

### Data analysis and statistics

Statistical analysis is described in the supplementary data.

## Supplementary Information

Supplementary Information.

## Data Availability

The datasets used and/or analyzed during the current study available from the corresponding author on reasonable request.
