# Supplementary material for: Structure optimization of new tumor-selective Passerini α-acyloxy carboxamides as Caspase-3/7 activators
Source: Sci Rep. 2022 Dec 27;12:22390. doi: 10.1038/s41598-022-26469-4 (PMC9794698; doi:10.1038/s41598-022-26469-4)
Supplement: Supplementary file 1 — Supplementary Information. [file 41598_2022_26469_MOESM1_ESM.pdf]

Structure optimization of new tumor-selective Passerini α-acyloxy carboxamides Caspase-3/7 activators

Mohammed Salah Ayoup [a] *, Yasmin Wahby [a], Hamida Abdel-Hamid [a], Marwa M. Abu-Serie [b], Mohamed Teleb [c]

[a] Chemistry Department, Faculty of Science, Alexandria University, P.O. Box 426, Alexandria, 21321, Egypt.
[b] Medical Biotechnology Department, Genetic Engineering and Biotechnology Research Institute, City of Scientific Research and Technological Applications (SRTA-City), Egypt.
[c] Department of Pharmaceutical Chemistry, Faculty of Pharmacy, Alexandria University, Alexandria, 21521, Egypt.
*: Corresponding Author: mohammedsalahayoup@gmail.com & Mohamed.salah@alexu.edu.eg

---

Contents **Page**

1. Figure S1 IR of compound 4 **S2**
2. Figures S2-S23 ^{1}HNMR and ^{13}CNMR for compounds **7a-7k** **S3-S13**
3. Figure S24, S25 and ^{1}HNMR and ^{13}CNMR for compounds **9** **S14**
3. Figure S26, S27 ^{1}HNMR and ^{13}CNMR for compounds **11** **S15**
4. Materials and Equipment **S16**
5. *Biological evaluation* **S16**
5.1. MTT assay **S16**
5.2. Caspase-3/7 activation assay **S17**
5.3. Real-time Quantitative PCR Analysis of Bcl2 **S17**
5.4. Flow cytometric analysis of apoptosis **S17**
5.5. Data analysis and statistics **S18**
6. References **S18**

1. IR

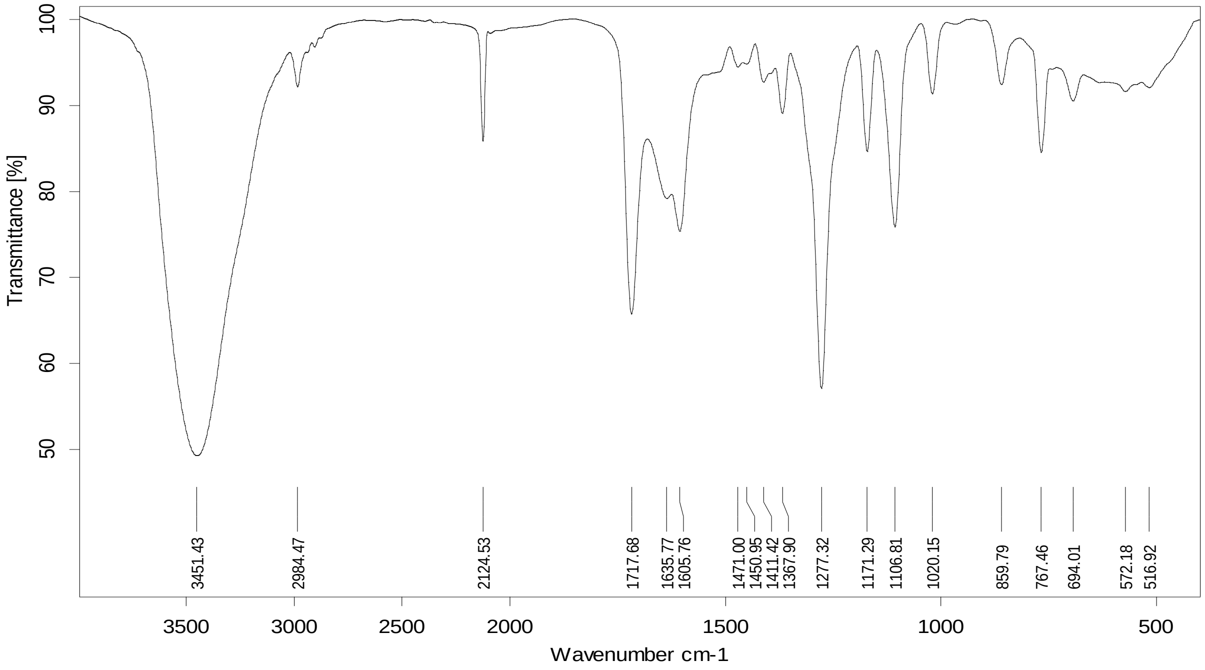

Figure S1. IR of 4

2. ^{1}HNMR and ^{13}CNMR of compounds **7a-k**

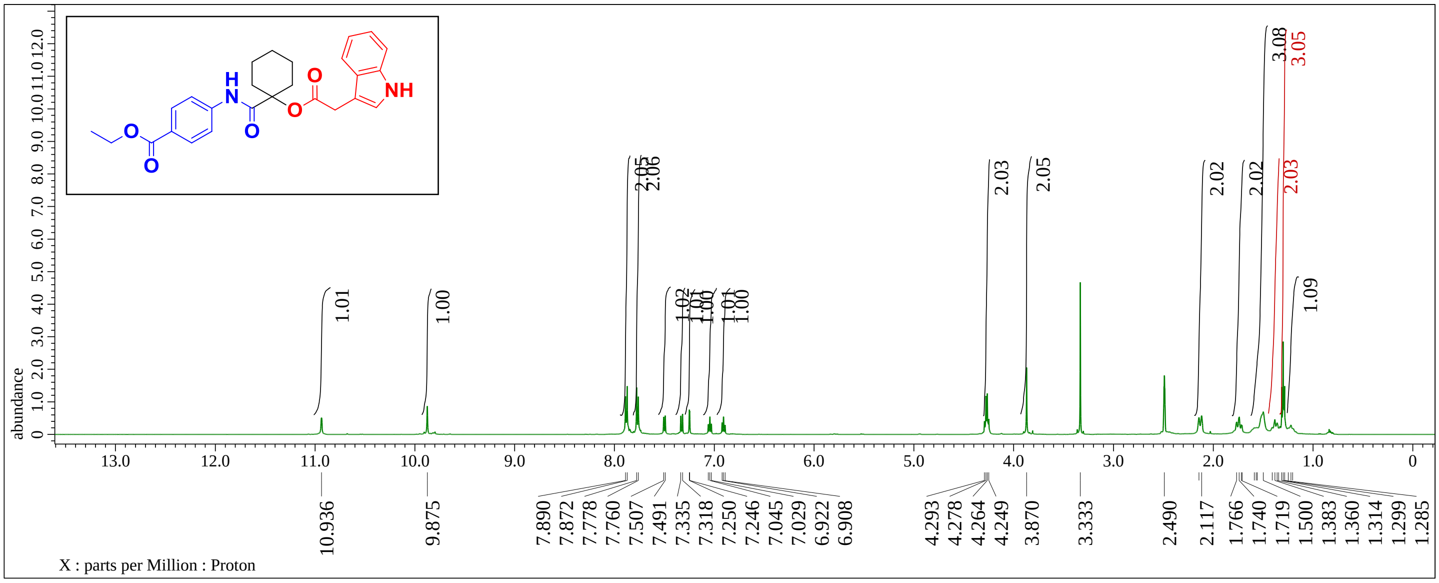

Figure S2. ^{1}H-NMR of 7a (DMSO-d_6)

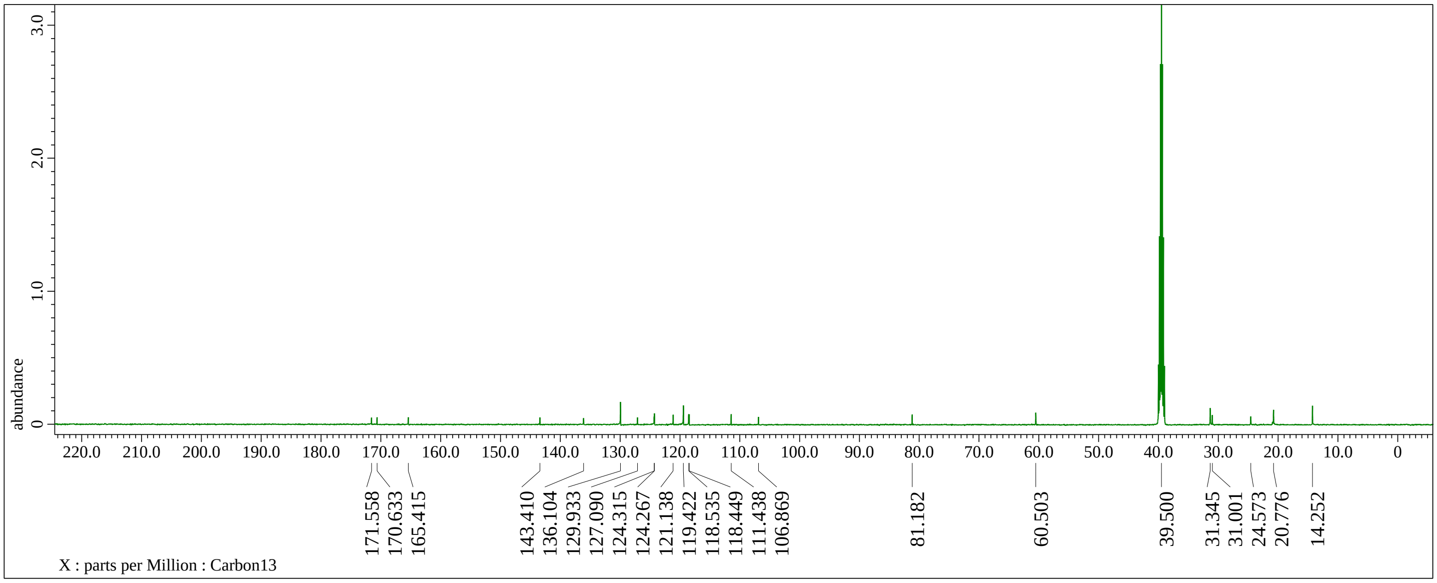

Figure S3. ^{13}C-NMR of 7a (DMSO-d_6)

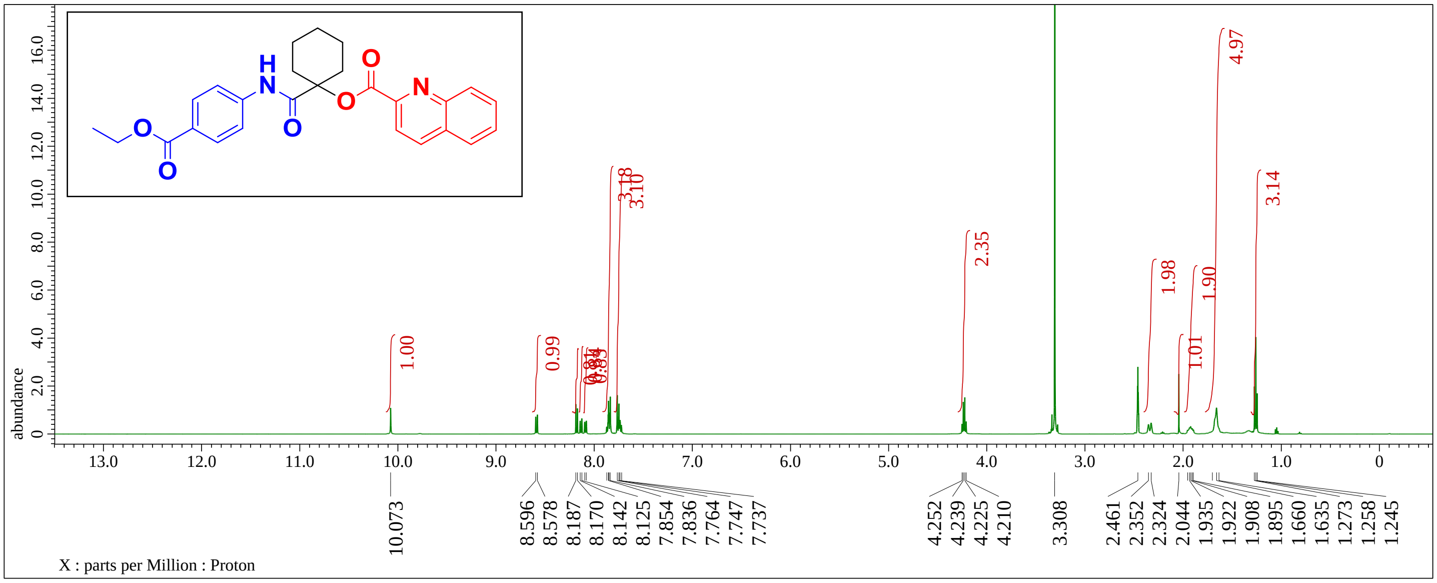

Figure S4. ^{1}H-NMR of 7b (DMSO-d_6)

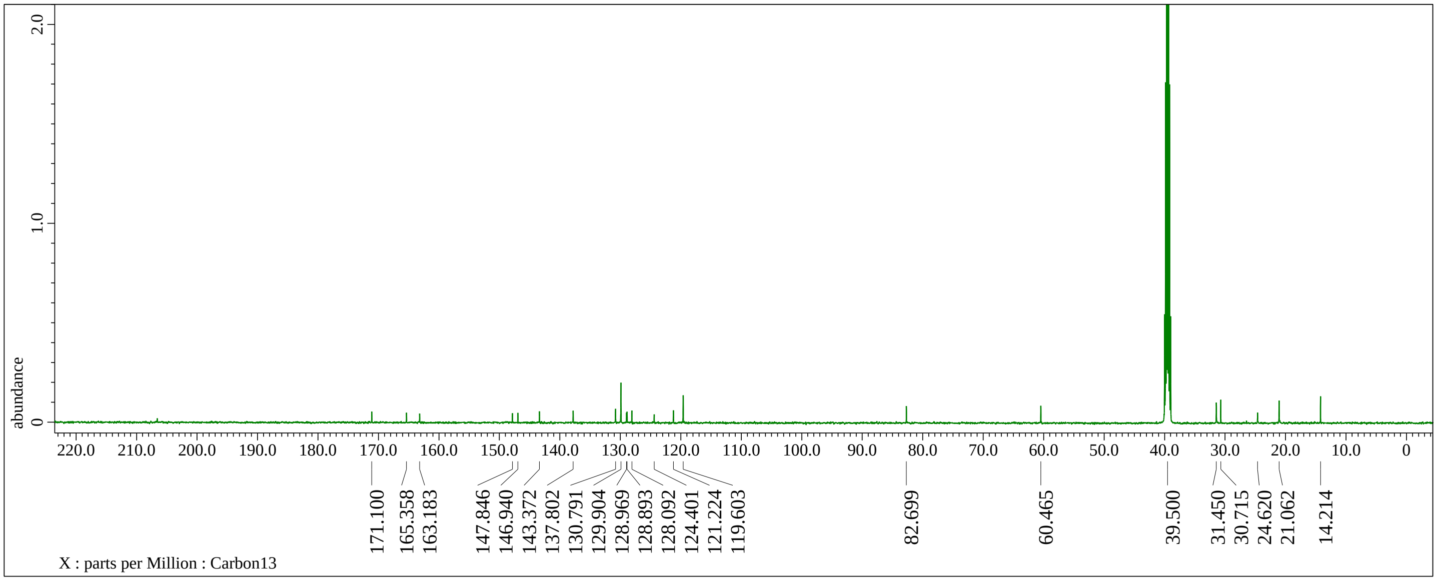

Figure S5. ^{13}C-NMR of 7b (DMSO-d_6)

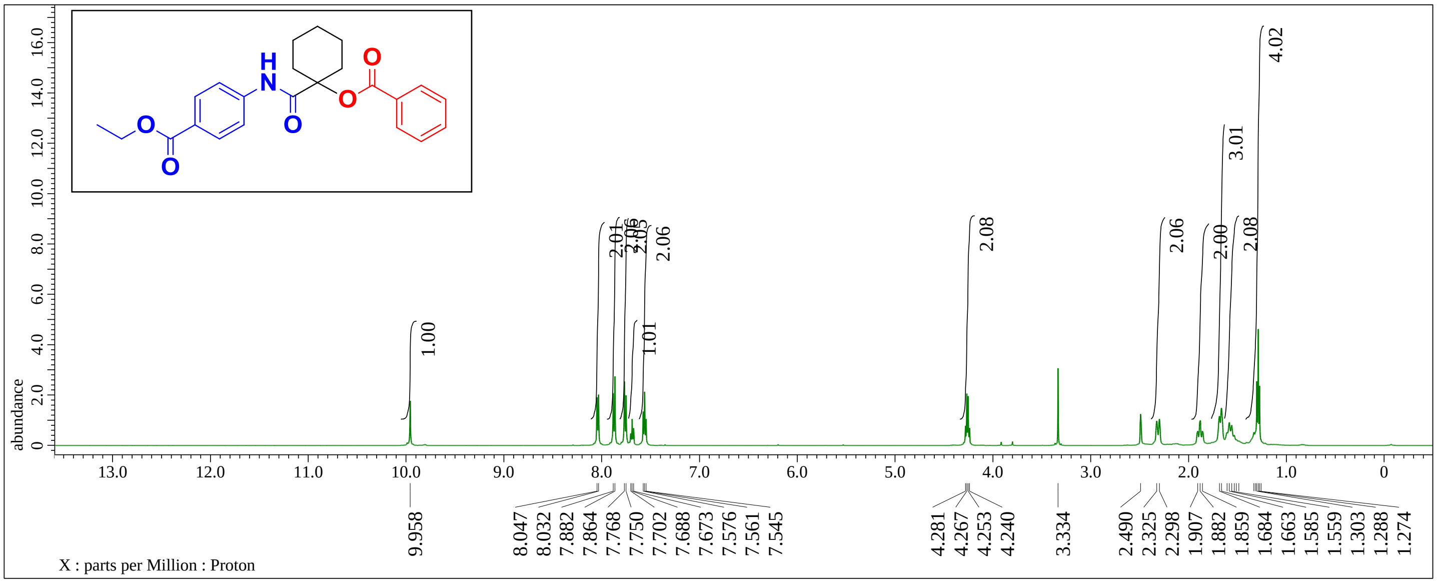

Figure S6. ^{1}H-NMR of 7c (DMSO-d_6)

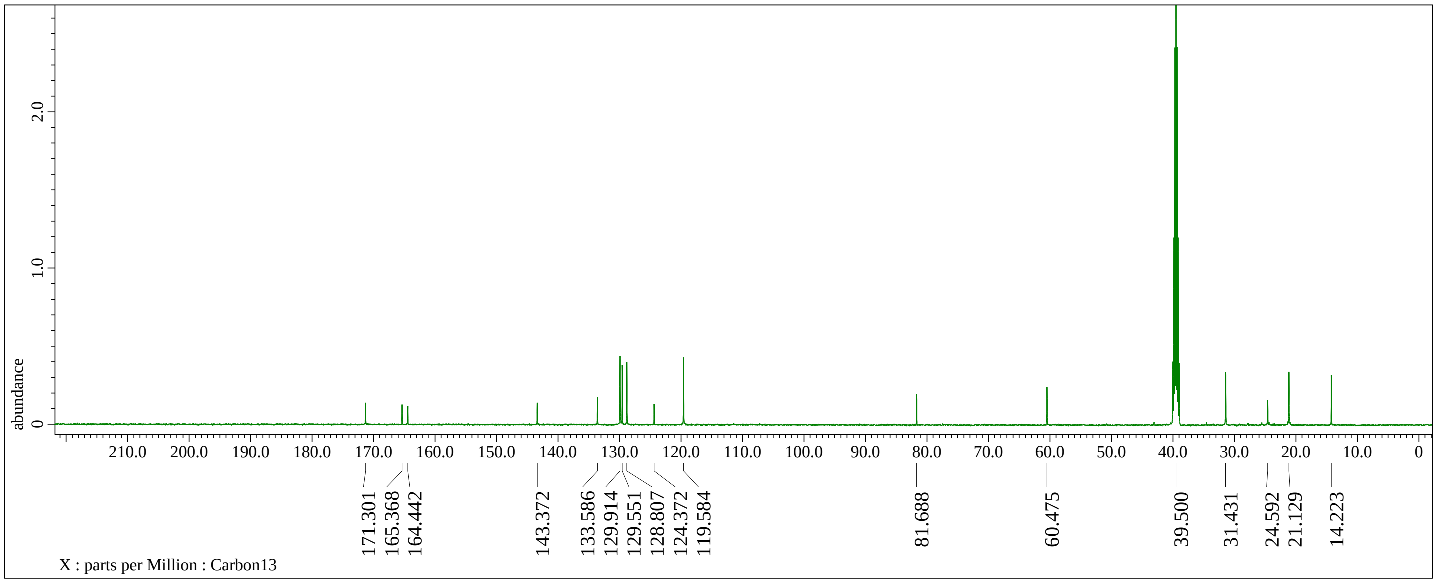

Figure S7. ^{13}C-NMR of 7c (DMSO-d_6)

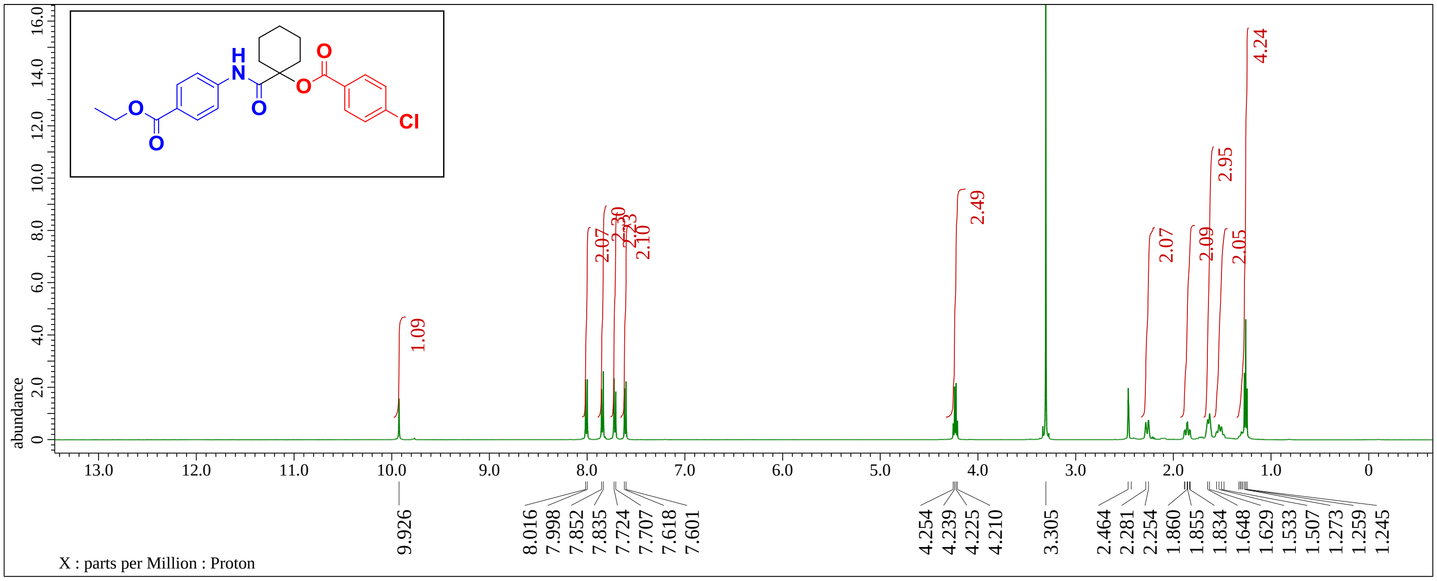

Figure S8. ^{1}H-NMR of 7d (DMSO-d_6)

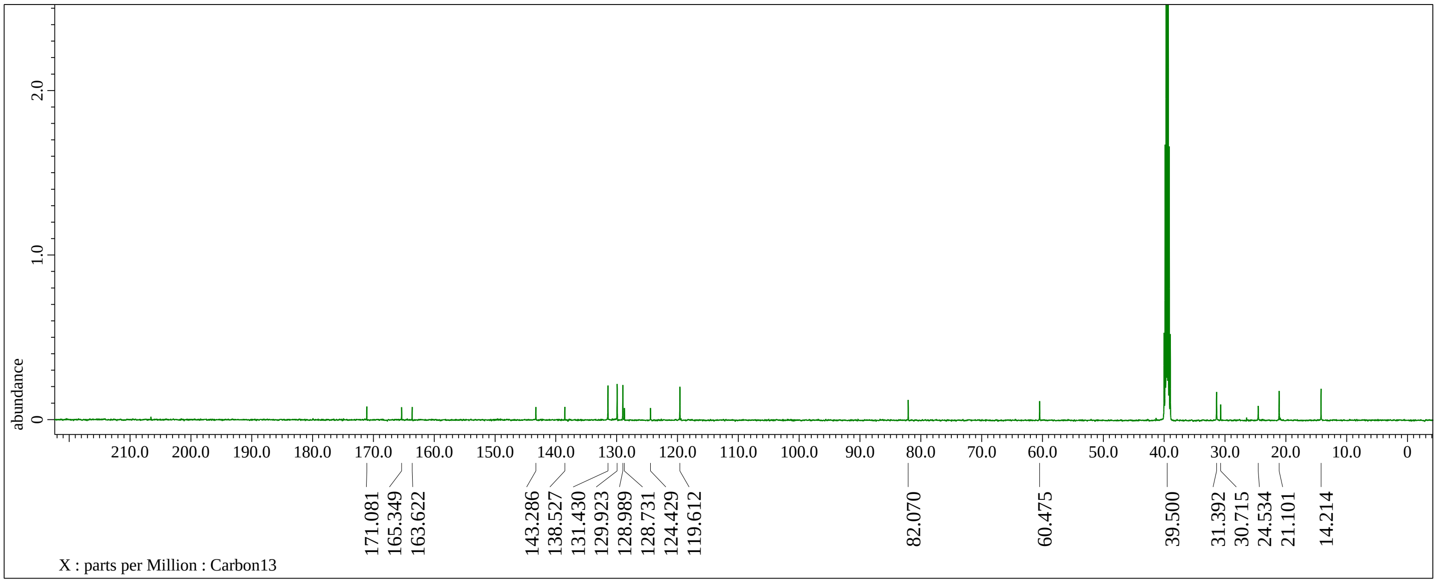

Figure S9. ^{13}C-NMR of 7d (DMSO-d_6)

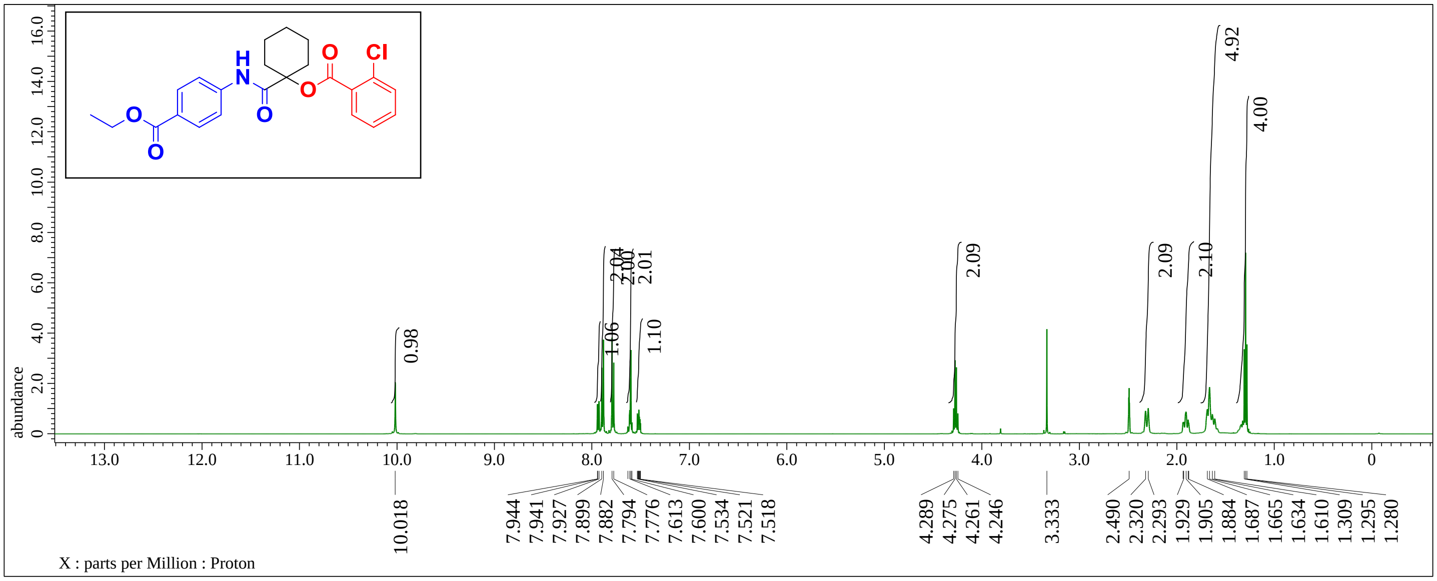

Figure S10. ^{1}H-NMR of 7e (DMSO-d_6)

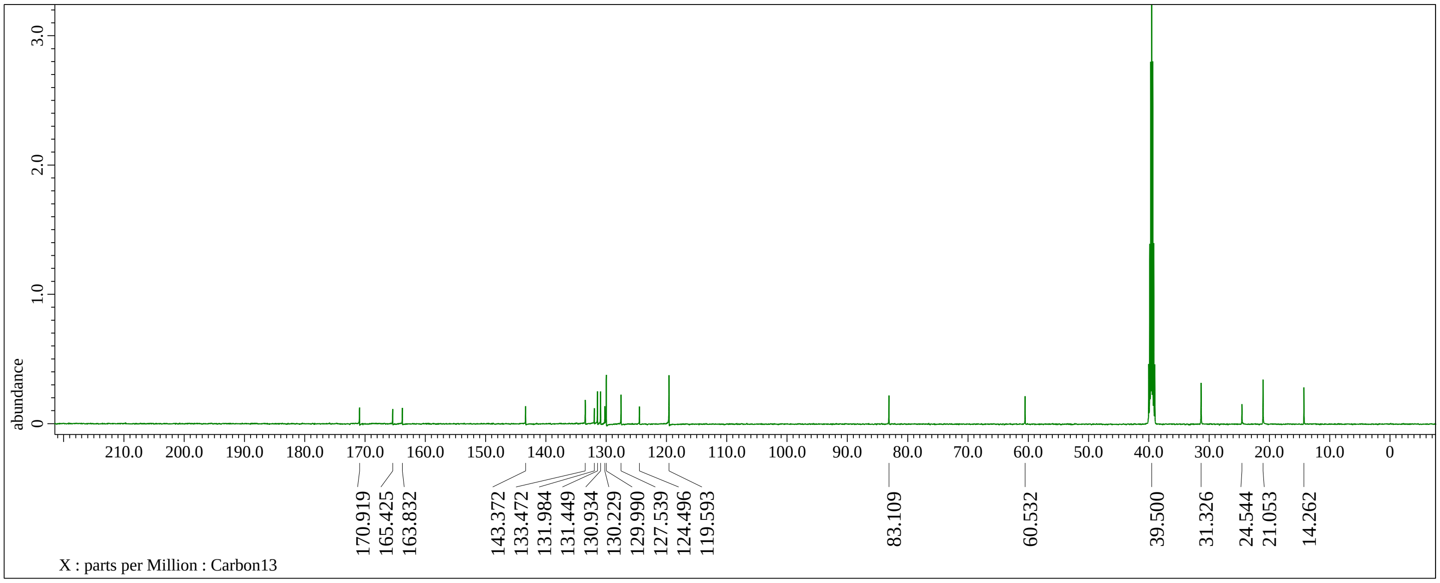

Figure S11. ^{13}C-NMR of 7e (DMSO-d_6)

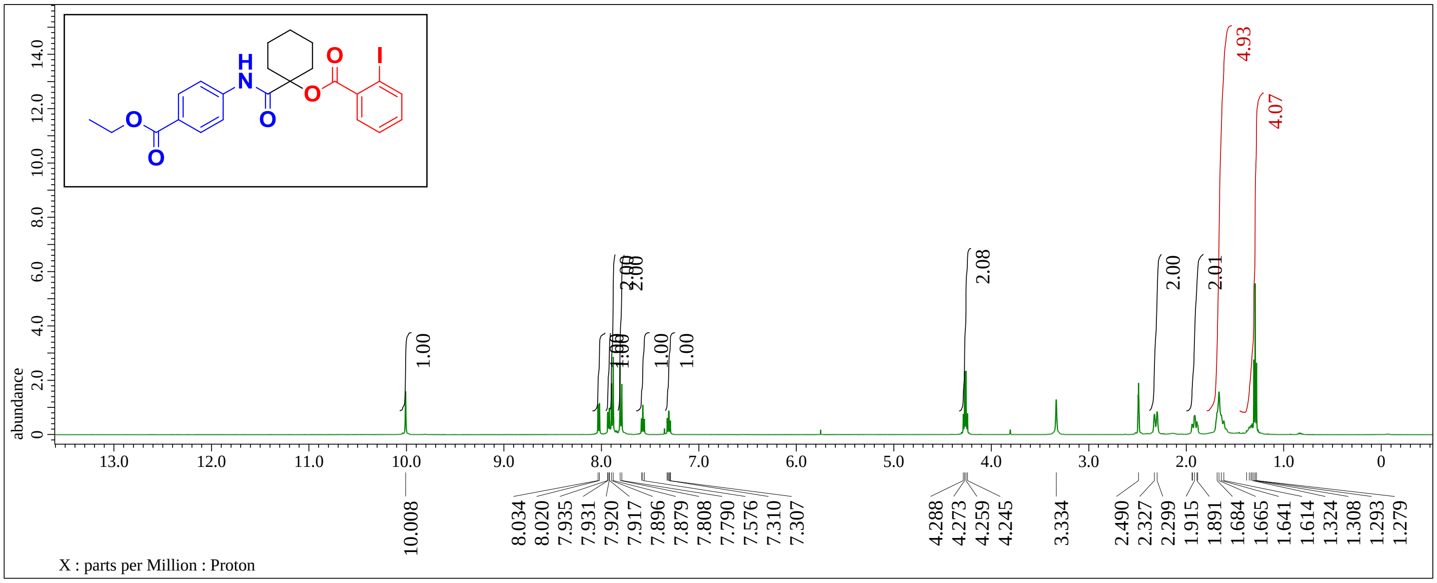

Figure S12. ^{1}H-NMR of 7f (DMSO-d_6)

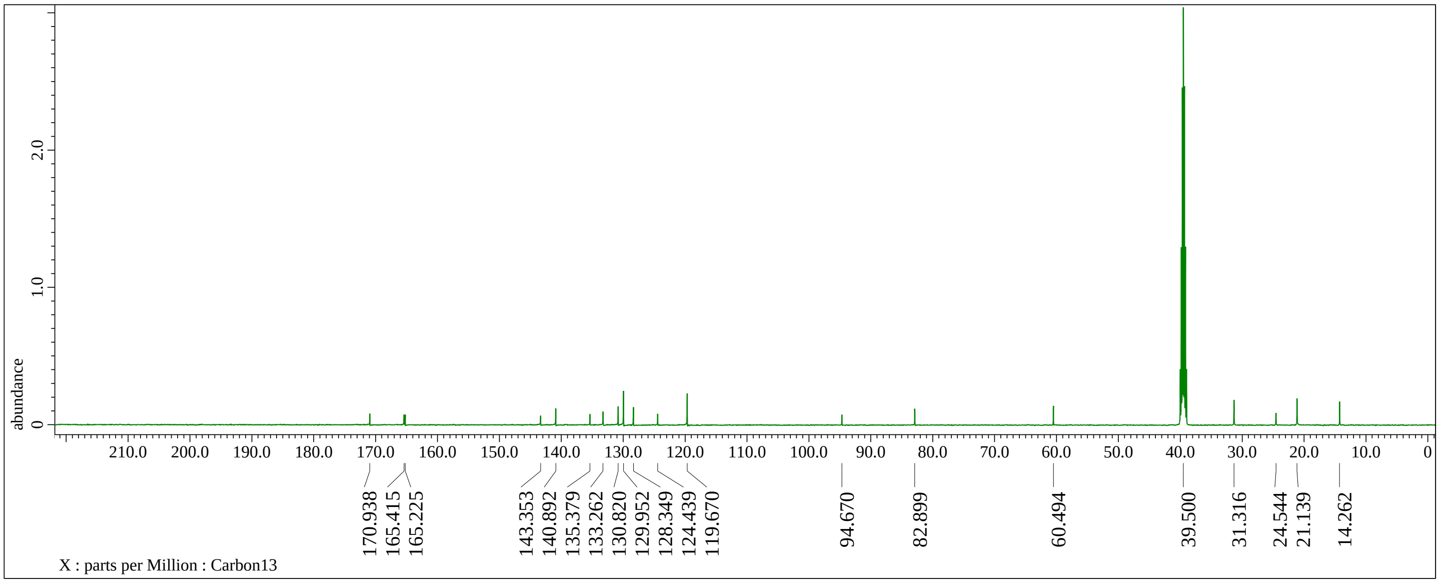

Figure S13. ^{13}C-NMR of 7f (DMSO-d_6)

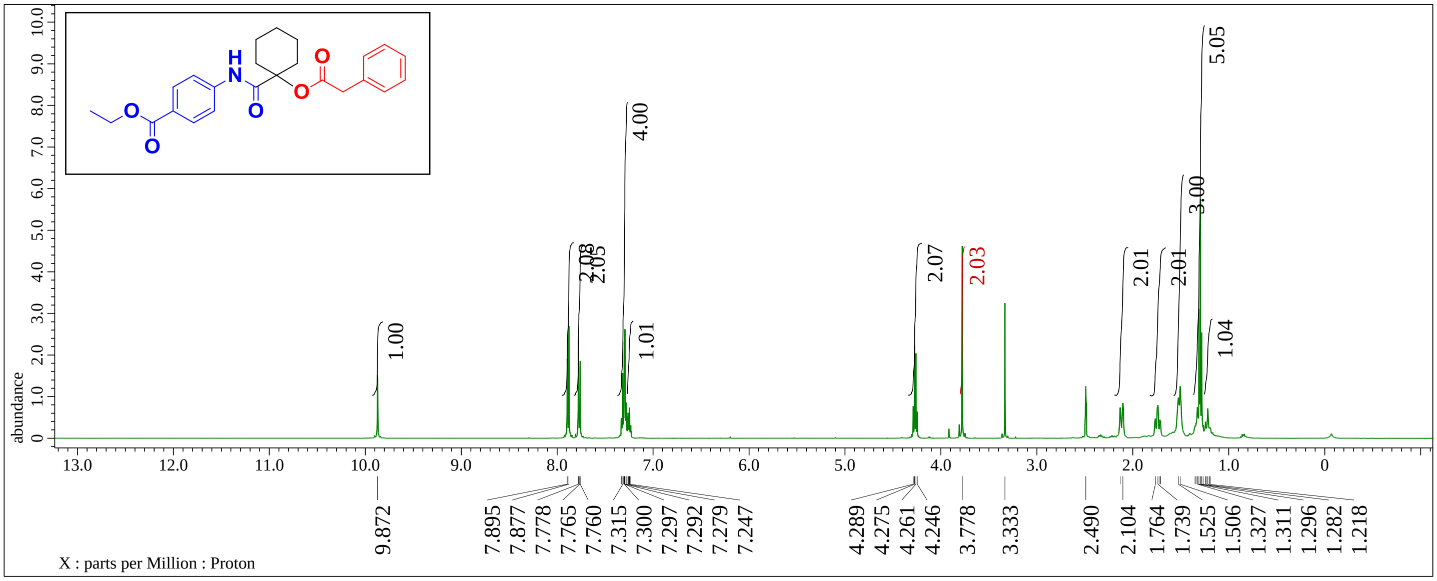

Figure S14. ^{1}H-NMR of 7g (DMSO-d_6)

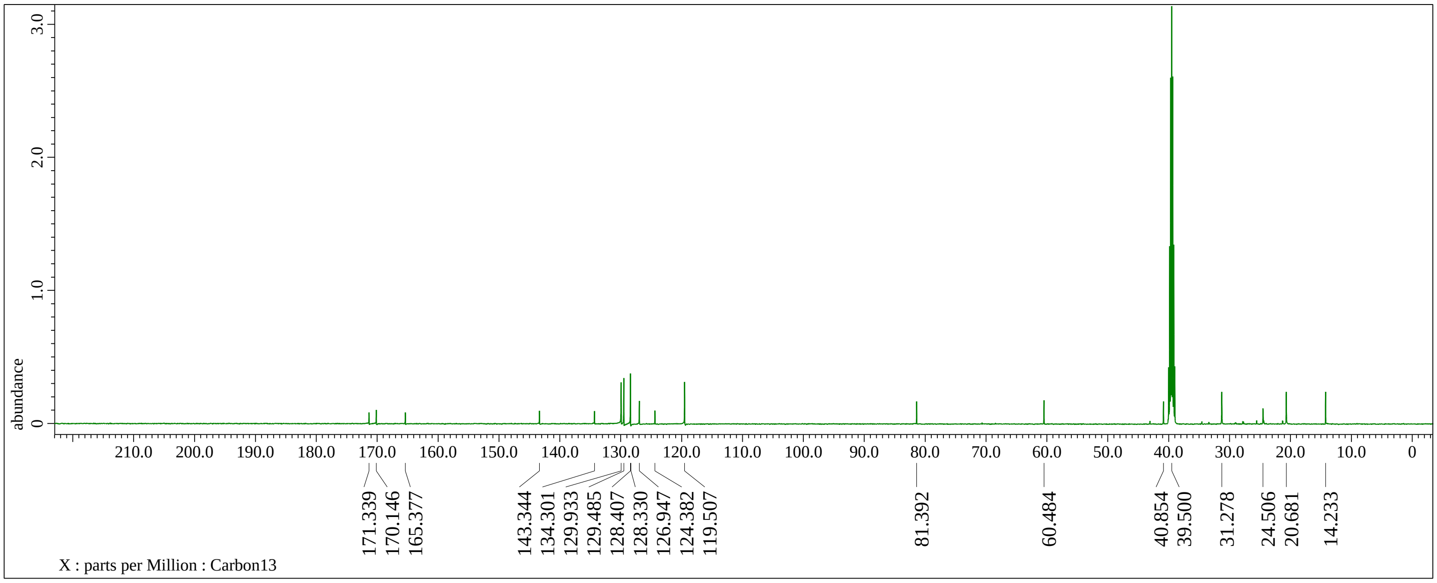

Figure S15. ^{13}C-NMR of 7g (DMSO-d_6)

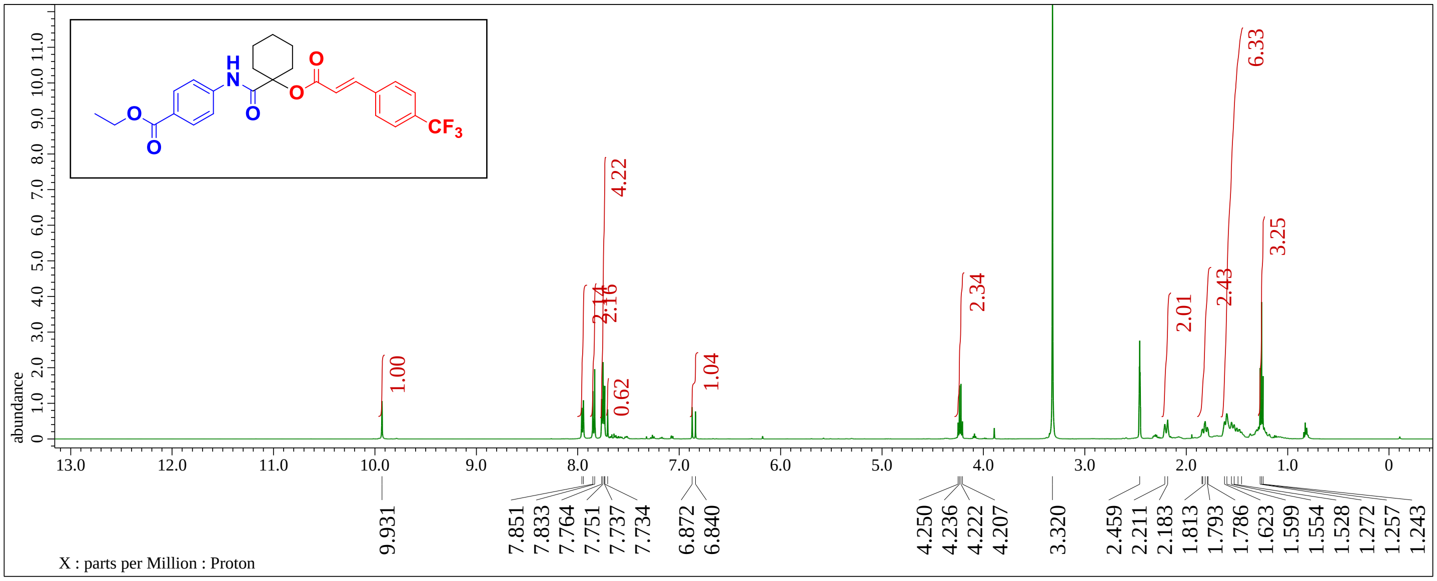

Figure S16. ^{1}H-NMR of 7h (DMSO-d_6)

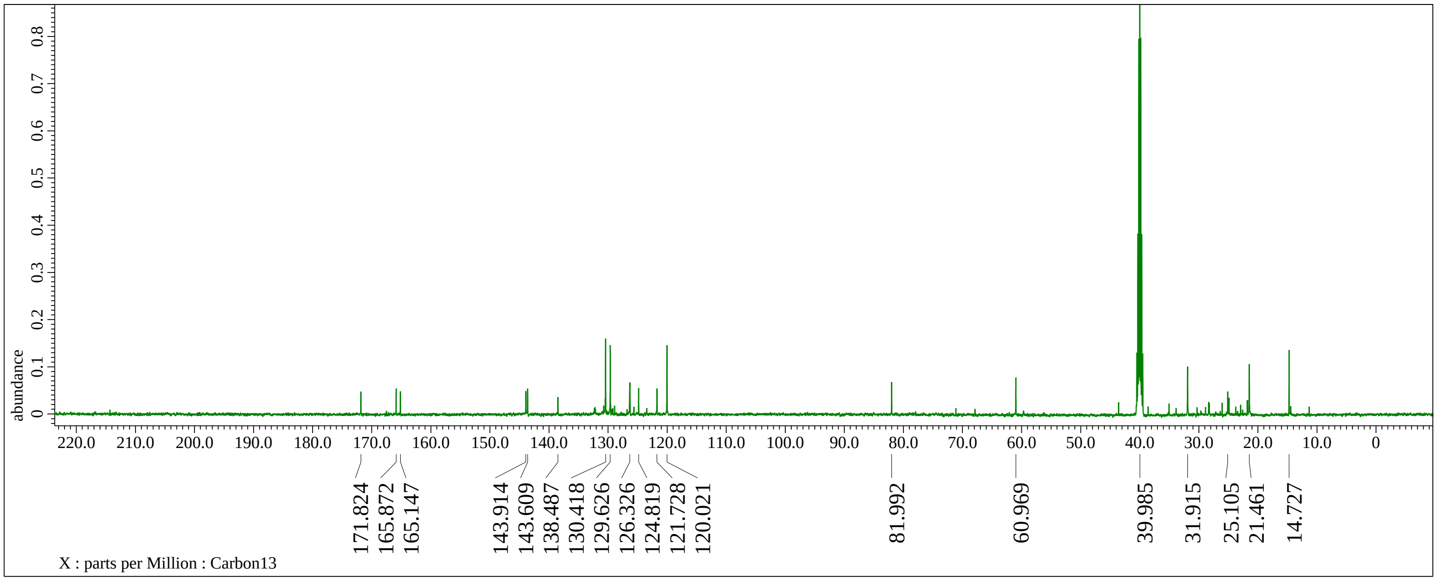

Figure S17. ^{13}C-NMR of 7h (DMSO-d_6)

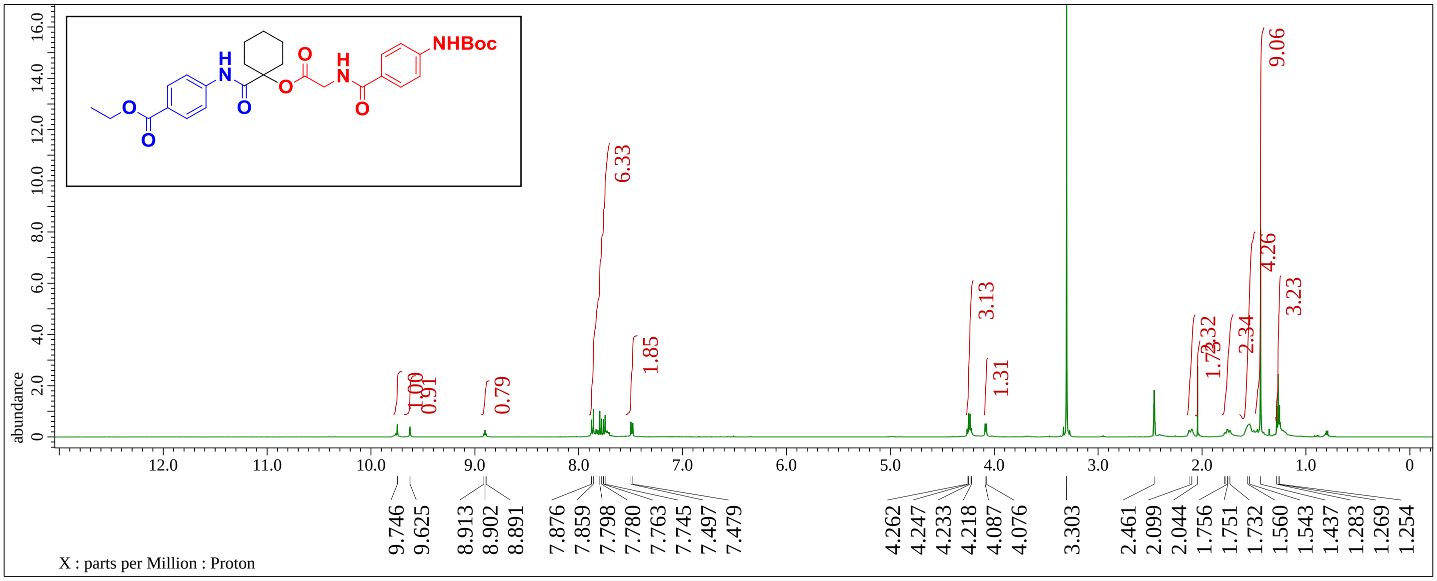

Figure S18. ^{1}H-NMR of 7i (DMSO-d_6)

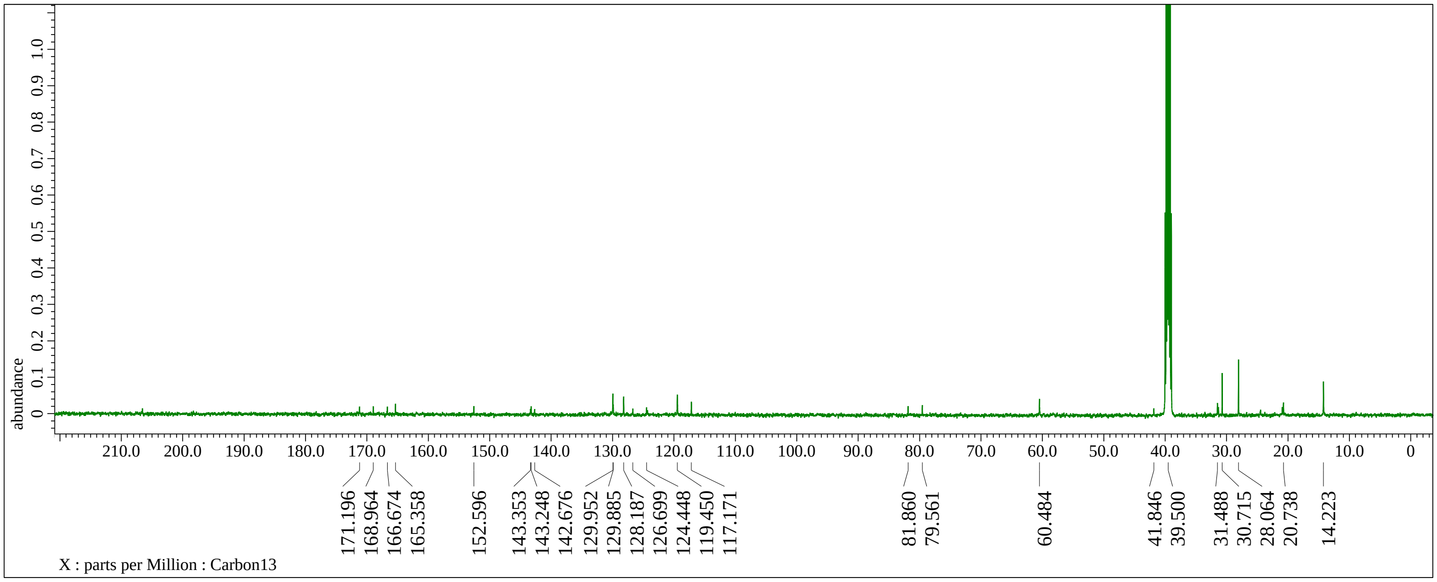

Figure S19. ^{13}C-NMR of 7i (DMSO-d_6)

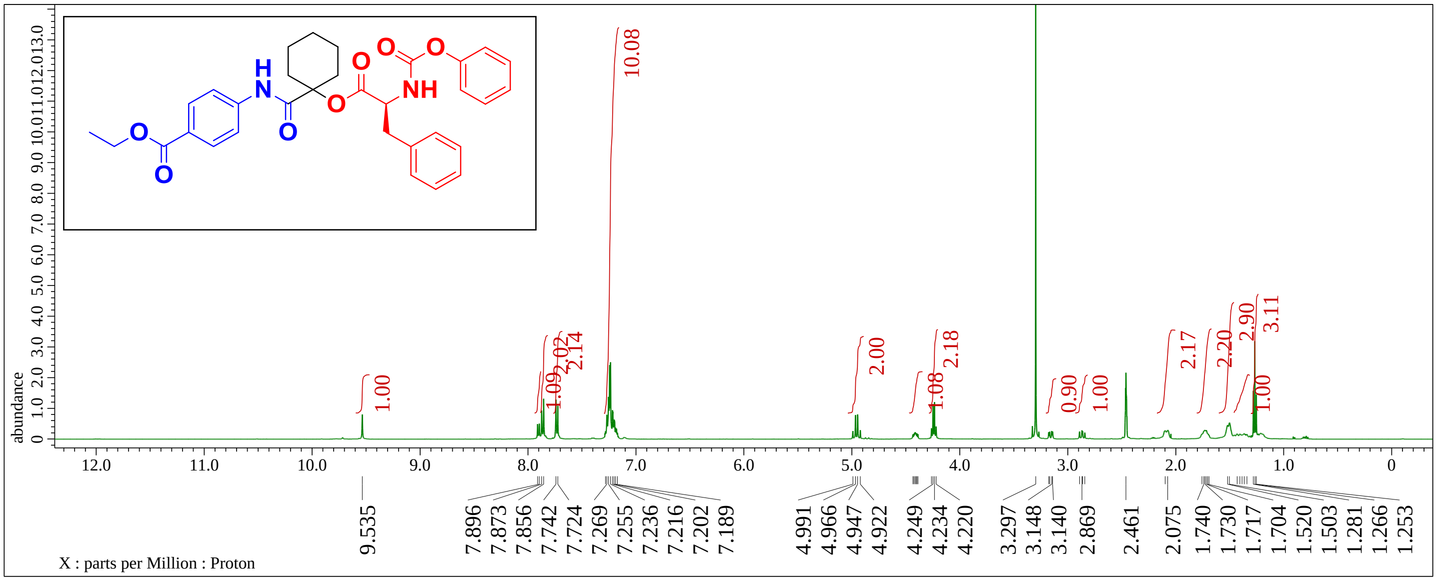

Figure S20. ^{1}H-NMR of 7j (DMSO-d_6)

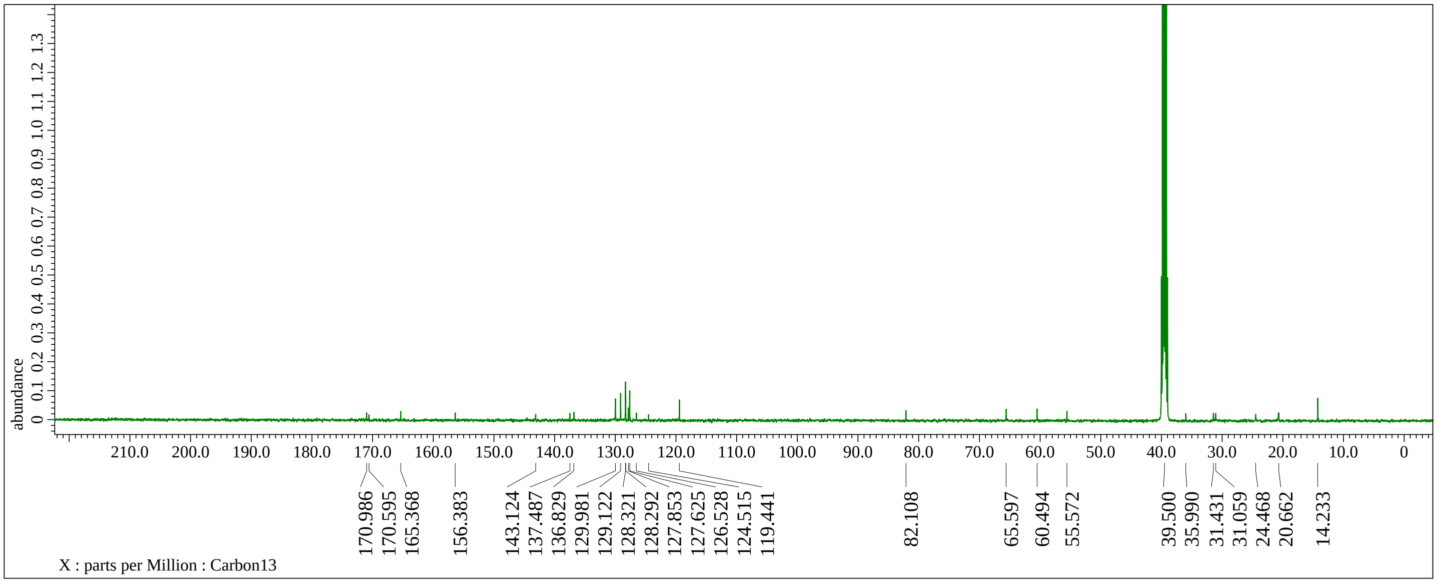

Figure S21. ^{13}C-NMR of 7j (DMSO-d_6)

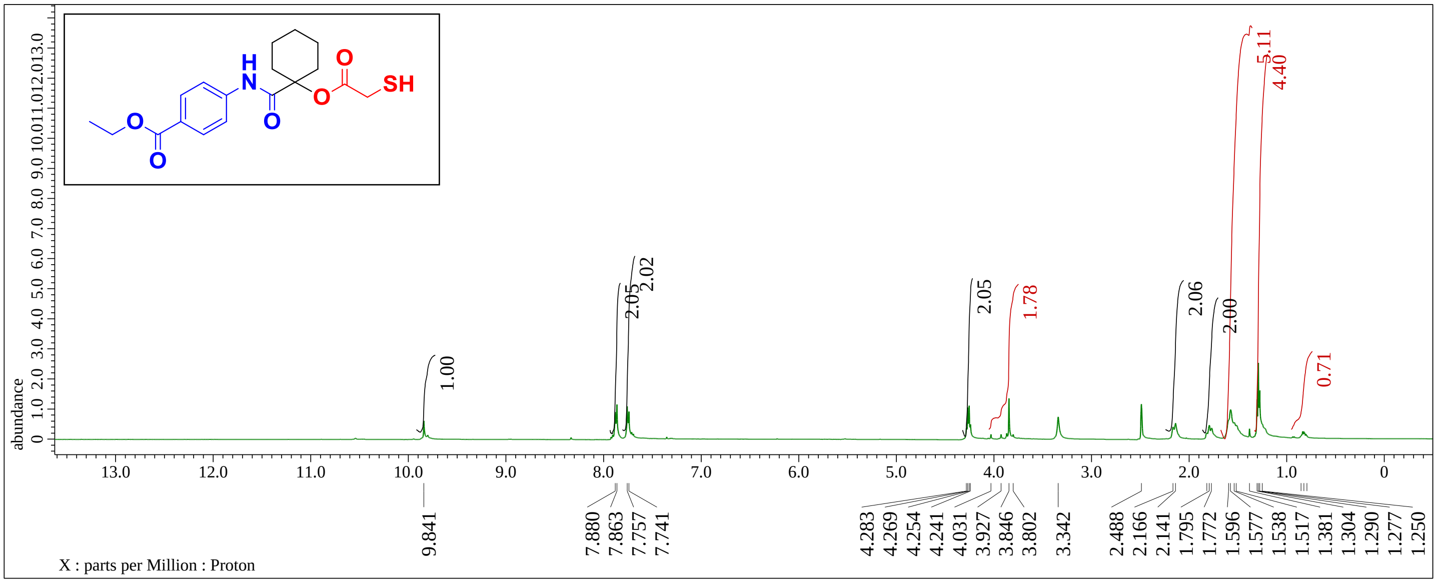

Figure S22. ^{1}H-NMR of 7k (DMSO-d_6)

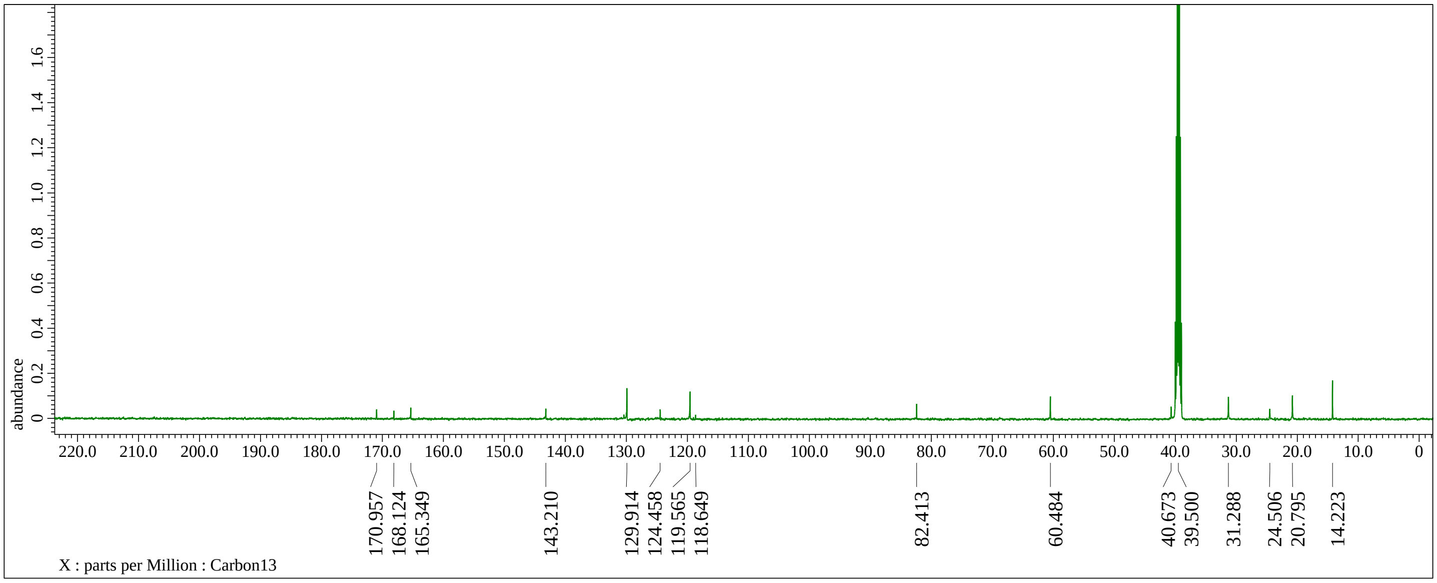

Figure S23. ^{13}C-NMR of 7k (DMSO-d_6)

3. ^{1}HNMR and ^{13}CNMR of compounds **9**

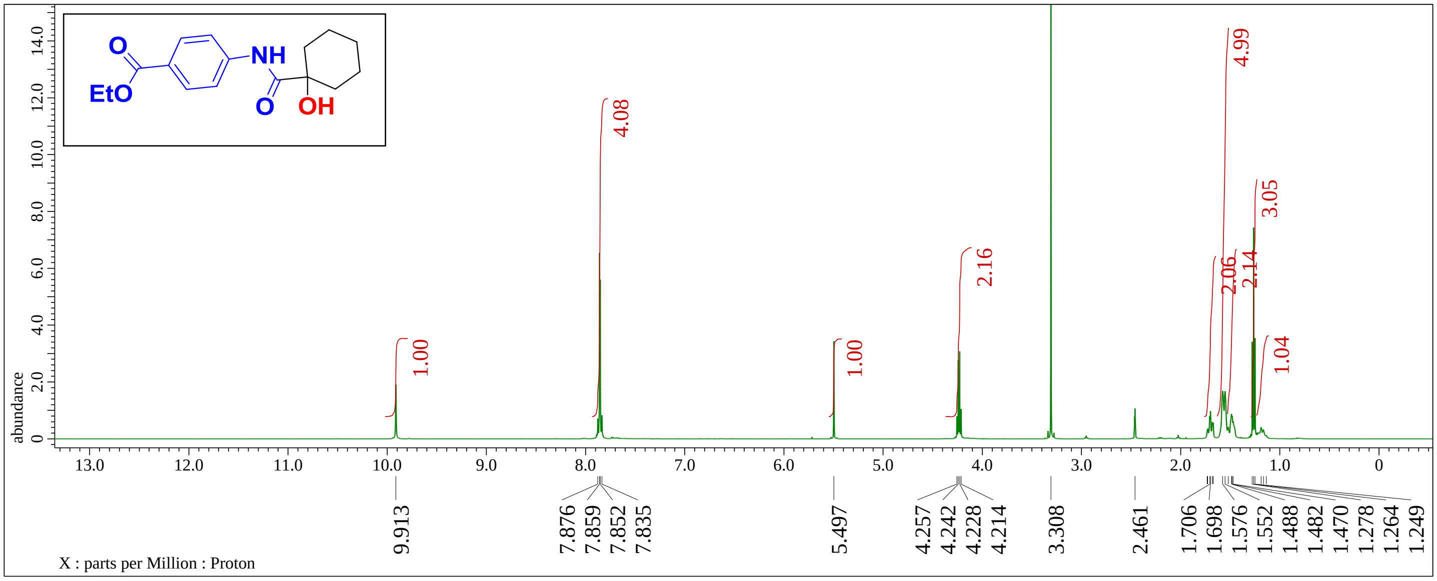

Figure S24. ^{1}H-NMR of 9 (DMSO-d_6)

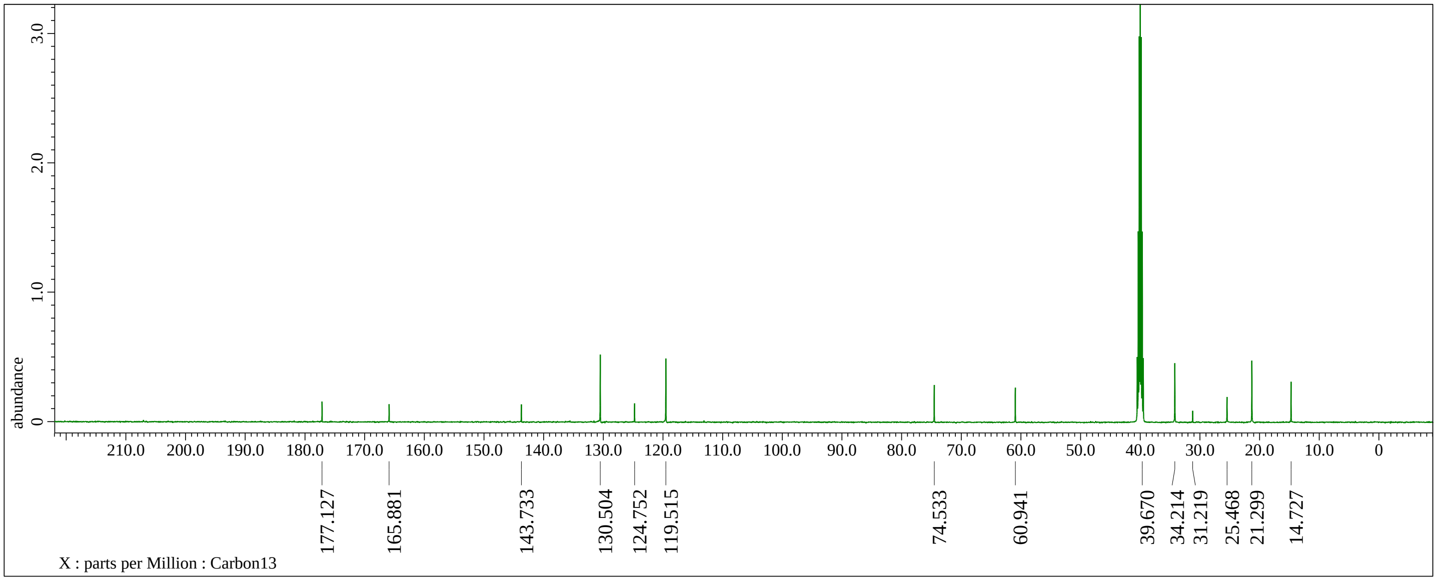

Figure S25. ^{13}C-NMR of 9 (DMSO-d_6)

4. ^{1}HNMR and ^{13}CNMR of compounds **11**

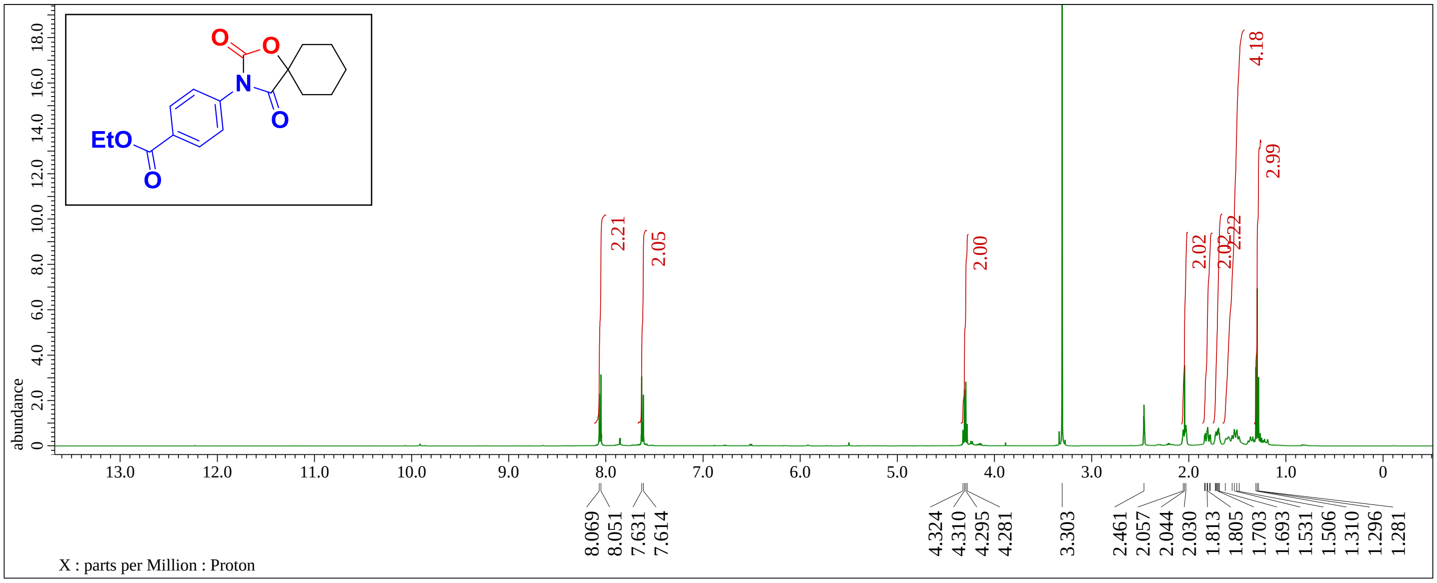

Figure S26. ^{1}H-NMR of 11 (DMSO-d_6)

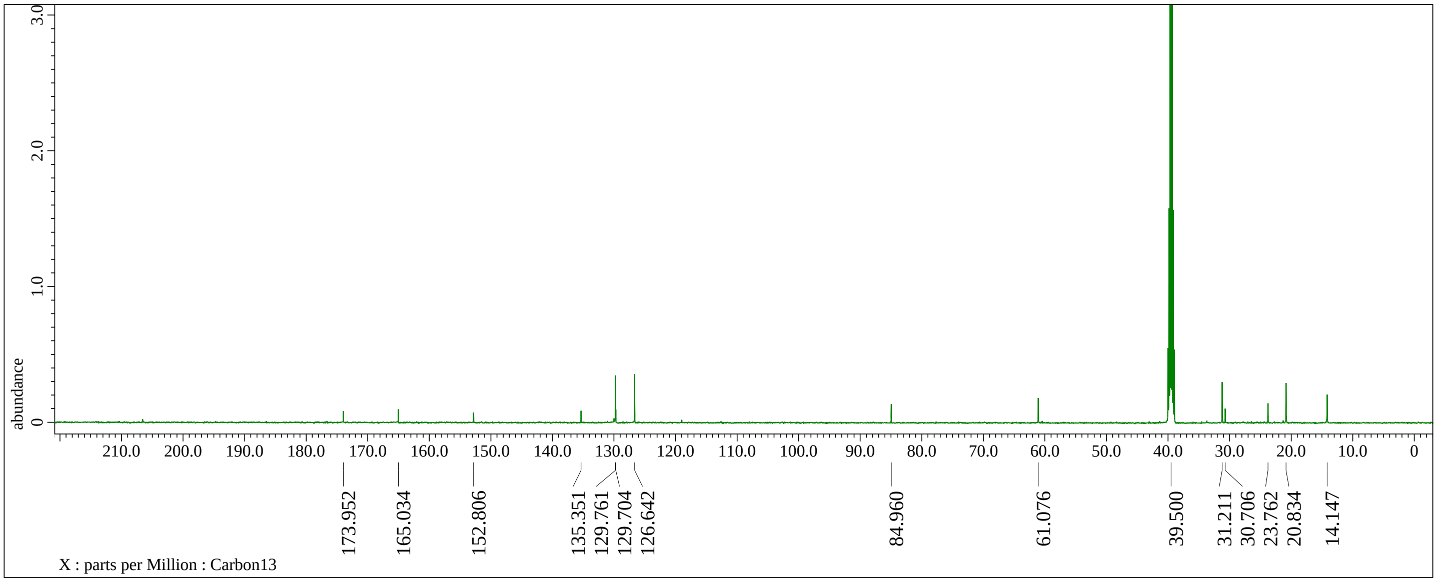

Figure S27. ^{13}C-NMR of 11 (DMSO-d_6)

4. Materials and Equipment.

Commercially available solvents and reagents were purified according to the standard procedures. Dried glassware was used for all reactions. ^{1}H-NMR data were recorded by JEOLJNM ECA 500 MHz. The deuterated solvent was used as an internal deuterium lock. ^{13}C-NMR spectra were measured using 125 MHz UDEFT pulse sequence and broad-band proton decoupling. Chemical shifts were reported in parts per million (δ/ppm). Internal reference for NMR spectra is tetramethylsilane at 0.00 ppm. Coupling constants are recorded in Hertz (J/Hz). IR [ν/cm^{-1}] data were reported using Perkin Elmer; FT-IR Spectrum BX and Bruker tensor 37 FT-IR. Thin layer chromatography (TLC) was performed on silica gel plastic plates and the spots were indicated under UV VILBER LOURMAT 4w-265nm or 254 nm tube. Melting points were measured by Thermo Scientific, Model NO. 1002D; 220-240v; 200W; 50/60Hz and were uncorrected.

5. Biological evaluation

5.1. MTT assay

Normal human lung fibroblast Wi-38 cell line was used to detect cytotoxicity of the studied compounds. Wi-38 cell line was cultured in DMEM medium-contained 10% fetal bovine serum (FBS), seeded as 5x10^{3} cells per well in 96-well cell culture plate and incubated at 37ºC in 5% CO_2 incubator. After 24 h for cell attachment, serial concentrations of the synthetic compounds and standard chemotherapy (5-fluorouracil) were incubated with Wi-38 cells for 72 h. Cell viability was assayed by MTT method [1] Twenty microliters of 5 mg/ml MTT (Sigma, USA) was added to each well and the plate was incubated at 37ºC for 3 h. Then MTT solution was removed, 100 µl DMSO was added, and the absorbance of each well was measured with a microplate reader (BMG LabTech, Germany) at 570 nm. The dose (EC50 and EC_{100}) values (at 50% and100% cell viability, respectively) of the tested compounds was estimated by the Graphpad Instat software.

The anticancer effect of the above-mentioned compounds was assayed using human colon cancer cell line (Caco-2) and liver cancer cell line (HepG-2) that were cultured in DMEM (Lonza, USA) supplemented with 10% FBS. All cancer cells (5x10^{3} cells/well) were seeded in sterile 96-well plates. After 24h, serial concentrations of the tested compounds and 5-fluorouracil (Fu) were incubated with two cancer cell lines for 72 h at 37ºC in 5% CO_2 incubator. MTT method was done as described above. The half maximal inhibitory concentration (IC_{50}) values were calculated using the Graphpad Instat software. Furthermore, cellular morphological changes before and after treatment with the most effective and safest anticancer compounds were investigated using phase contrast inverted microscope with a digital camera (Olympus, Japan).

5.2. Caspase-3/7 activation assay

The caspase 3/7 activation was assessed, as a key indicator in caspase-dependent apoptosis, using ApoONE® Caspase-3/7 kit following the manufacturer's instructions. This kit used a nonfluorescence substrate that was cleaved by caspases of the most active-treated cancer cells, relative to the untreated cells, resulting in the generation of the fluorescence signals of Rhodamine 110. Thesesignal were measured at 490 nm excitation and 520 nm emission using the fluorescence microplate reader (BMG LabTech, Germany).

5.3. Real-time Quantitative PCR Analysis of Bcl2

Colon cancer cell line (Caco-2) was incubated with the most effective anticancer compounds, at 0.06 µM, for 72 h in 5% CO_2 incubator. RNAs of untreated and treated cancer cells were extracted using Gene JET RNA purification kit (Thermo Scientific, USA). Then cDNAs were synthesized using cDNA Synthesis Kit (Thermo Scientific, USA). Real time PCR was performed using SYBR green master mix and specific primers (Forward/Reverse) as shown in Table 1. The thermal cycling parameters were pre-denaturation, followed by 40 amplification cycles of 1 min at 95°C and 30 s at 60 °C and at 72°C for 30 sec. GAPDH mRNA was quantified to adjust the amount of mRNA in each sample. The $2^{-\Delta\Delta CT}$ equation was used to estimate change in gene expressions before and after treatment of cancer cells [2,3].

Table 1 primer sequences of the used genes

Gene	Primers
BCl2	Forward: 5′-CTGGTGGACAACATCGCCCT-3′<br>Reverse: 5′-TCTTCAGAGACAGCCAGGAGAAAT-3′

5.4. Flow cytometric analysis of apoptosis

The most effective compounds were selected to investigate their proapoptotic effect by incubation with human colon cancer cell line (Caco-2) for 72 h, at their minimum IC_{50} doses. After trypsinization, the untreated and treated cancer cells were stained with fluorescein isothiocyanate (FITC)-annexin V/ propidium iodide (PI) for 15 min. After washing with PBS, the annexin-stained apoptotic population was quantified using flow cytometry at FITC signal detector (FL1) against the phycoerythrin emission signal detector (FL2) [4].

5.5. Data analysis and statistics

Data were expressed as mean ± standard error of the mean (SEM). Statistical significance was estimated by the multiple comparisons Tukey post-hoc analysis of variance (ANOVA) using the SPSS16 program. The differences were considered statistically significant at $p < 0.05$

6. References

1- T. Mosmann, Rapid colorimetric assay for cellular growth and survival: application to proliferation and cytotoxicity assays, *J. Immunol. Methods* 65(1-2) (1983) 55-63.

2- Y. Li, L. Lin, Q. Wang, Correlation of expression levels of caspase-3 and Bcl-2 in alveolar lavage fluid in neonatal respiratory distress syndrome and prognosis, *Exp. Ther. Med.* 15(3) (2018) 2891-2895.

3- J.r. Grünenfelder, D.N. Miniati, S. Murata, V. Falk, E.G. Hoyt, M. Kown, M.L. Koransky, R.C. Robbins, Upregulation of Bcl-2 through caspase-3 inhibition ameliorates ischemia/reperfusion injury in rat cardiac allografts, *Circulation* 104 (2001),202-206

4- M.S. Ayoup, Y. Wahby, H. Abdel-Hamid, M. Teleb, M.M. Abu-Serie, A. Noby, Design, synthesis and biological evaluation of novel α-acyloxy carboxamides via Passerini reaction as caspase 3/7 activators, *Eur. J. Med. Chem.* 168 (2019) 340-356.
